# Assessing quality of life—a scoping review of studies presenting quality of life instruments for informal caregivers of persons with dementia

**DOI:** 10.1186/s12877-025-06455-x

**Published:** 2025-11-22

**Authors:** Daniela Lillekroken, Heidi Bjørge, Liv Halvorsrud, Ingeborg Beate Lidal

**Affiliations:** 1 Department of Nursing and Health Promotion, Oslo Metropolitan University, PB 4 St. Olavs plass, Oslo, N – 0130 Norway; 2 Norwegian Institute of Public Health, Oslo, Norway

**Keywords:** Dementia, Informal caregivers, Instruments, Quality of life, Scoping review

## Abstract

**Background:**

Assessing the quality of life in informal caregivers of people with dementia is crucial, as it may signal the need for support, counselling, and other care-related needs for their loved ones. Although various quality-of-life instruments are available, selecting an appropriate instrument suited to this population is a complex and challenging task. Therefore, this scoping review aims to identify and map the existing literature on instruments used to measure the quality of life of informal caregivers of people living with dementia.

**Method:**

Seven databases (Medline, Embase, PsycINFO, CINAHL, SocIndex, Web of Science, Epistemonikos) were searched for original, relevant, peer-reviewed articles in English published between May 2016 and January 2025. This scoping review adhered to international methodological guidelines and is presented in accordance with the Preferred Reporting Items for Systematic Reviews and Meta-Analyses extension for Scoping Reviews (PRISMA-ScR) checklist.

**Results:**

A total of 34 studies, reporting on 21 instruments used to assess quality of life in informal caregivers of people with dementia, are included. These comprise four generic instruments (CASP-16, SF-12, SF-36, and WHOQOL-BREF) and 17 disease- and population-specific instruments. The SF-36, the WHOQOL-BREF, and the C-DEMQOL were the most frequently used, appearing in five studies each. Overall, there was limited evidence regarding the instruments’ internal consistency and content validity, and the review results suggested that none stood out as superior for use with informal caregivers of people with dementia.

**Conclusion:**

Several instruments have been used to assess the quality of life in informal caregivers of people with dementia; however, few were specifically developed or validated for this population. No instrument emerged as clearly superior, and evidence on content validity and internal consistency was generally limited. Instruments tailored to caregivers, such as the APPLIQUE, ASCOT-Carer, LTCQ-Carer, and SCQOLS-D, showed greater relevance to caregiving experiences. At the same time, generic instruments like the WHOQOL-BREF often included less applicable items and lacked validation in this context. These findings emphasise the need for careful instrument selection, with a focus on relevance to caregiving domains, and highlight the importance of future research that includes qualitative input, caregiver involvement, and robust psychometric testing across diverse settings.

**PROSPERO registration:**

CRD42022327590.

**Supplementary Information:**

The online version contains supplementary material available at 10.1186/s12877-025-06455-x.

## Background

The World Health Organization (WHO) reports that over 55 million people worldwide are living with dementia, with nearly 10 million new cases appearing each year [1]. Dementia is a common condition in older age, characterised by a chronic, progressive, irreversible brain disorder that typically includes neuropsychiatric symptoms and mood changes, a decline in cognitive ability, and consequently, reduced functional capacity [1, 2]. Most people with dementia live at home [3]. As their ability to cope gradually diminishes, they require health and social care from both family members and healthcare services, which can be costly for all involved [4]. Relatives who provide care and support to a person with dementia are directly or indirectly affected by the condition, and the burden on informal caregivers can be overwhelming [5]. Therefore, several recent studies have been conducted to improve understanding of the experiences associated with informal caregiving [6–9].

Informal caregivers, typically family members such as spouses, children, daughters-in-law, or friends, most of whom are women [10], often take on primary responsibility for caring for people with dementia. This role often involves long hours and a broad range of care tasks that are not fully covered by formal support services [11]. As dementia advances, informal caregivers are required to provide increasingly complex and time-consuming care [5, 6]. These growing demands can negatively impact caregivers’ physical and mental health, especially concerning emotional stress and the changing nature of the caregiver-patient relationship [12]. Additionally, caregivers’ perceptions of their role and the dementia itself—shaped by their understanding of the illness, the support and information available at different stages, and feelings of unpreparedness—may deepen their sense of burden [13]. These mental health and psychological issues form a crucial basis for understanding how caregiving burden affects wider outcomes, particularly caregivers’ quality of life (QoL). Given this burden, it is not surprising that several studies [14, 15] have reported a negative effect on perceived QoL among informal caregivers of people with dementia.

Measuring QoL in informal caregivers of people with dementia is an important outcome and a proxy for caregiver sustainability and wellbeing, as it reflects the multidimensional impact of these demands on caregivers’ lives [12–14]. Another reason for assessing QoL is to evaluate the effectiveness of interventions aimed at informal caregivers, which may indirectly reduce caregiver burden, stress, and anxiety [17] and improve their physical and psychological health outcomes [18]. Consequently, changes in caregiver QoL can act as a crucial indicator of intervention success. Additionally, QoL may assist in planning timely and suitable support services [19]. Given these points, QoL remains a key outcome and a proxy for caregiver sustainability and wellbeing.

The WHO Quality of Life assessment group [16] defines QoL as ‘an individual’s perception of their position in life in the context of the culture and value systems in which they live and concerning their goals, expectations, standards and concerns’ [16, p. 1405]]. This definition highlights the subjective nature of QoL, which is directly affected by the caregiver’s health, emotional state, and perceived level of support, factors previously discussed. As a result, research increasingly seeks to measure informal caregivers’ QoL using various instruments, reflecting the growing recognition of its importance in caregiving settings [12, 14, 20–23]. Recognising that Qol is based on an individual’s personal experience, research methods for measuring QoL have expanded [24, 25]. Understanding and improving informal caregivers’ QoL is vital, not only for their well-being but also for sustaining the caregiving relationship. Hence, clinicians and researchers are encouraged to undertake early screening and interventions to lower health risks associated with reduced QoL [26]. Despite this, there is no consensus on the best method to assess QoL [27]. Many instruments have been developed to evaluate QoL in both healthy and ill individuals. When choosing appropriate instruments, alignment with conceptual frameworks, clear definitions, and the intended purpose are essential to achieve meaningful results [27, 28].

Instruments are designed to include relevant variables specific to a particular population group. They can consist of one or multiple domains and items that can be scored either as a total score or divided into subdomains [24]. According to Mokkink et al. [29], instruments must be selected carefully, considering the quality of the instrument, patient- reported outcome measures (PROMs), relevance and comprehensiveness, the analysis process, and the purpose of measuring QoL. The quality of instruments is commonly described in terms of various aspects of validity and reliability [29–32]. Farina et al. [12] have systematically reviewed existing outcome measures and emphasised the importance of internal consistency for accurately assessing the Qol of informal caregivers. Mokkink et al. [29] propose that content validity is the most crucial measurement property because the items of a PROM must be relevant, comprehensive, and understandable for the construct being measured and for the target population. Therefore, when evaluating the content validity of an instrument measuring QoL in informal caregivers of people with dementia, it is vital to assess whether the included items are relevant to the construct, suitable for the target population, and appropriate within the context of use. It is also important to ensure that response options and recall periods are suitable. A key part of this process involves confirming that the QoL domains covered by the instrument are meaningful from the perspective of informal caregivers themselves. Ideally, this entails directly asking informal caregivers whether the instrument captures the aspects of QoL that matter most to them. Furthermore, it is essential to verify that no key concepts are overlooked, that the items and response options are clearly and appropriately worded, and that instructions, items, and response scales are understood by informal caregivers as intended. Instruments measuring QoL in informal caregivers of people with dementia should include unidimensional scales to guarantee valid, interpretable, and actionable results. The absence of unidimensionality can undermine the usefulness of the instrument in both research and clinical practice. Therefore, ensuring the internal consistency of instruments is vital for obtaining reliable and valid results, especially for instruments or subscales presumed to be unidimensional [34]. QoL instruments often include distinct domains (e.g., emotional well-being, social functioning, burden), each typically designed to reflect a single underlying construct. Internal consistency measures, such as Cronbach’s alpha, are commonly used, with a value of 0.70 often cited as an acceptable reliability threshold. However, Cronbach’s alpha should be interpreted with caution: higher values can arise from item redundancy or very narrow constructs, while broader constructs or shorter scales may produce lower values despite being theoretically and practically sound. In some cases, shorter instruments may even be preferable due to reduced participant burden. Moreover, Cronbach’s alpha is only one way to assess internal consistency; other coefficients, such as McDonald’s omega, might provide more accurate estimates, especially when the assumptions of tau-equivalence are violated.Therefore, when selecting a QoL instrument, it is important to evaluate its internal consistency to ensure a dependable assessment of informal caregivers’ experiences, which can, in turn, support the evaluation of interventions and healthcare services for informal caregivers of people with dementia.

QoL instruments are classified as overall, generic, and disease-specific [22]. An overall instrument may include a single question about informal caregivers’ perception of their QoL [22, 24]. Typically, an overall instrument comprises several dimensions that are combined to produce a total score. The number of items within each dimension should be considered before aggregating [27]. Generic instruments encompass health profiles and instruments that generate health utilities [22, 27]. They are recommended when assessing an individual’s overall health status or interventions that affect the individual as a whole [33]. Furthermore, generic instruments allow for comparisons between populations with different health issues and conditions [35]. Disease-specific instruments focus on aspects related to a particular disease state, patient group, or areas of physical or mental function [22, 36].

Regarding dementia care-related instruments, researchers have debated what might constitute the most relevant content, offering various suggestions [37]. Therefore, an instrument designed for informal caregivers of people with dementia should include the specific features of dementia and the impact of the chronic condition on caregivers, such as their own physical, mental, and social challenges [37]. However, although the research literature recommends using disease- and population-specific instruments when measuring QoL in a particular group, several generic QoL instruments are utilised in studies of informal caregivers of people with dementia, including the 12-item Short Form Health Survey (SF-12) [23], the 36-item Short Form Health Survey (SF-36) [38, 39], the European Quality of Life 5-Dimension questionnaire (EQ-5D) [21], the European Organisation for Research and Treatment of Cancer Quality of Life Questionnaire (EORTC QLQ-C30) [40], and the World Health Organization Quality of Life-BREF (WHOQOL-BREF) [41*]. In addition to these generic instruments, other instruments are also employed, such as the Measurement of Quality of Life in Family Carers of People with Dementia (C-DEMQOL) [42] and the Caregiver-Targeted Quality of Life measure (CGQOL) [43].

The challenges faced by informal caregivers of people with dementia in their caregiving roles are well-documented in several systematic reviews [11, 44, 45]. It has also been claimed that many QoL instruments have been evaluated for these caregivers [20, 42, 46], and selecting appropriate measures has been identified as an ongoing issue [20]. At the same time, it is important to avoid overburdening informal caregivers with unnecessary self-report questionnaires. However, limited information is available on the most commonly used instruments or which one to choose for assessing QoL in these caregivers. Therefore, greater clarity is needed regarding the existing instruments used to evaluate QoL in informal caregivers of people with dementia. This scoping review aims to identify and map the instruments used to measure QoL in informal caregivers of people with dementia.

## Methods

A scoping review methodology was employed and reported in accordance with the PRISMA-ScR Checklist, developed by members of the Joanna Briggs Institute (JBI) and collaborating centres [47, 48] (Supplementary File 1). The five methodological steps for conducting a scoping review, as proposed by the JBI Manual [49], were followed.

The scoping review method was selected to map the scope of studies that used QoL instruments administered to informal caregivers of people with dementia, summarising the available evidence objectively, identifying knowledge gaps, and contributing to improved practice and future research. Given the purpose of conducting a scoping review, no critically appraised or synthesised answer to the research question is provided; the goal is to offer evidence of the particular phenomenon [48], specifically to identify and map the instruments used to measure QoL in informal caregivers of individuals with dementia. The scoping review protocol was registered with the International Prospective Register for Systematic Reviews (PROSPERO) to enhance transparency and credibility.

### Step 1: defining the research question

The research question guiding this scoping review is: Which instruments are used to assess QoL in informal caregivers of people with dementia?

### Step 2: identifying relevant studies

The Population Concept and Context framework (PCC) [48] has been used as a guide to develop clear and meaningful objectives and eligibility criteria for this scoping review. Therefore, the inclusion criteria were established considering the research question, with the assistance of the PCC framework. In this review, the population of interest was informal caregivers of people with dementia, and the concept was QoL and QoL instruments.

Studies were included if they: (i) presented one or multiple self-administered QoL instruments, (ii) were used for QoL assessment in informal caregivers of people with dementia, (iii) reported at least one psychometric property of the instrument used to measure QoL, and (iv) were published in English or a Scandinavian language between May 2016 and January 2025. The focus of this review was the home environment. Studies were excluded if they only reported the use or application of an instrument without providing at least one psychometric property of the instrument measuring QoL in informal caregivers of people with dementia.

Two systematic reviews conducted by Dow et al. [20] and Page et al. [33] on QoL instruments did not reach a consensus on which instrument offers the best standard for content and scoring. Our database searches showed that the review by Dow et al. [20], which included studies from April 2016, is the most recent publication discussing and evaluating instruments for measuring QoL in informal caregivers of people with dementia. To ensure the evidence reflects recent developments, May 2016 was chosen as the starting point for including articles in the current review, updating the evidence that used instruments measuring QoL in informal caregivers of people with dementia after Dow et al.’s [20] systematic review was published.

### Step 3: selection of studies

Two university librarians conducted a systematic search on 16 February 2021 to identify relevant studies in the following databases: Medline, Embase, PsycINFO, SocIndex, Social Care Online, CINAHL, Web of Science, and Google Scholar. The keywords or medical subject headings (MeSH) used by each database to index literature were searched within the titles, abstracts, and full texts of studies using the Boolean operators “AND”/”OR”.

The following keywords or MeSH terms and their synonyms were used to conduct a systematic search of the published literature: “informal caregiver” OR “family caregiver”, OR “caregivers”, “child”, “son/daughter”, “sibling”, “brother/sister”, “wife”, “husband”, “partner”, “spouse”, “married family”, “parent”, “father”, “mother”, “next of kin”, “significant other”, “friends”, “relative”, “informal”, “unpaid care”, “caring”, “caregiving”, “care giving”, AND “dementia”, “senile”, “Alzheimer”, OR “cognitive impairment”, AND “quality of life instrument”, “scale index”, “questionnaire”, “survey”, “test”, “scheme”, “tool”, “scoring”, AND “quality of life”, “life satisfaction”.

Due to the substantial time needed for article assessment and writing, the literature search was updated twice, first on 16 February 2024 and again on 9 January 2025. The revised database search identified 1,638 additional studies for evaluation, of which 18 were included in the current scoping review. A complete list of the updated search strategy as of 9 January 2025 is provided in Supplementary File 2.

### Step 4: mapping of information

After removing duplicate citations, all identified articles (citations and abstracts) were imported into the EndNote bibliographic manager [50]. Subsequently, the articles were transferred into Rayyan, a web tool (Beta) (http://rayyan.qcri.org) designed to assist researchers working on systematic reviews, scoping reviews, and other knowledge synthesis projects [51] for screening, in accordance with the PRISMA flow diagram [52].

The review process involved two levels of screening: first, the titles and abstracts, followed by a full-text review of relevant publications. The authors formed two pairs of reviewers, each consisting of two researchers (HB/IBL and LH/DL), who independently screened articles by title and abstract. The studies were evaluated based on the inclusion and exclusion criteria. Those meeting the inclusion criteria were read in full and reviewed for final eligibility by each pair. Disagreements on the eligibility of articles were resolved by involving the other pair of reviewers and discussing the matter until a consensus was reached.

According to Petters et al. [48], a scoping review does not involve assessing the methodological quality of the studies included. However, to improve transparency and clarity of the results, a descriptive quality review of all included studies was carried out. The same reviewers (HB/IBL and LH/DL) evaluated the studies for methodological validity using standardised critical assessment instruments from the JBI [53–56]. Discrepancies between reviewers’ assessments were resolved through discussion. If disagreements persisted, the other reviewers were consulted to reach a consensus. The outcomes of the quality review were not used to exclude studies but were considered during synthesis to highlight methodological trends and limitations in the field. An example of the quality review conducted for the cross-sectional studies is included in Supplementary File 3.

**Fig. 1:**
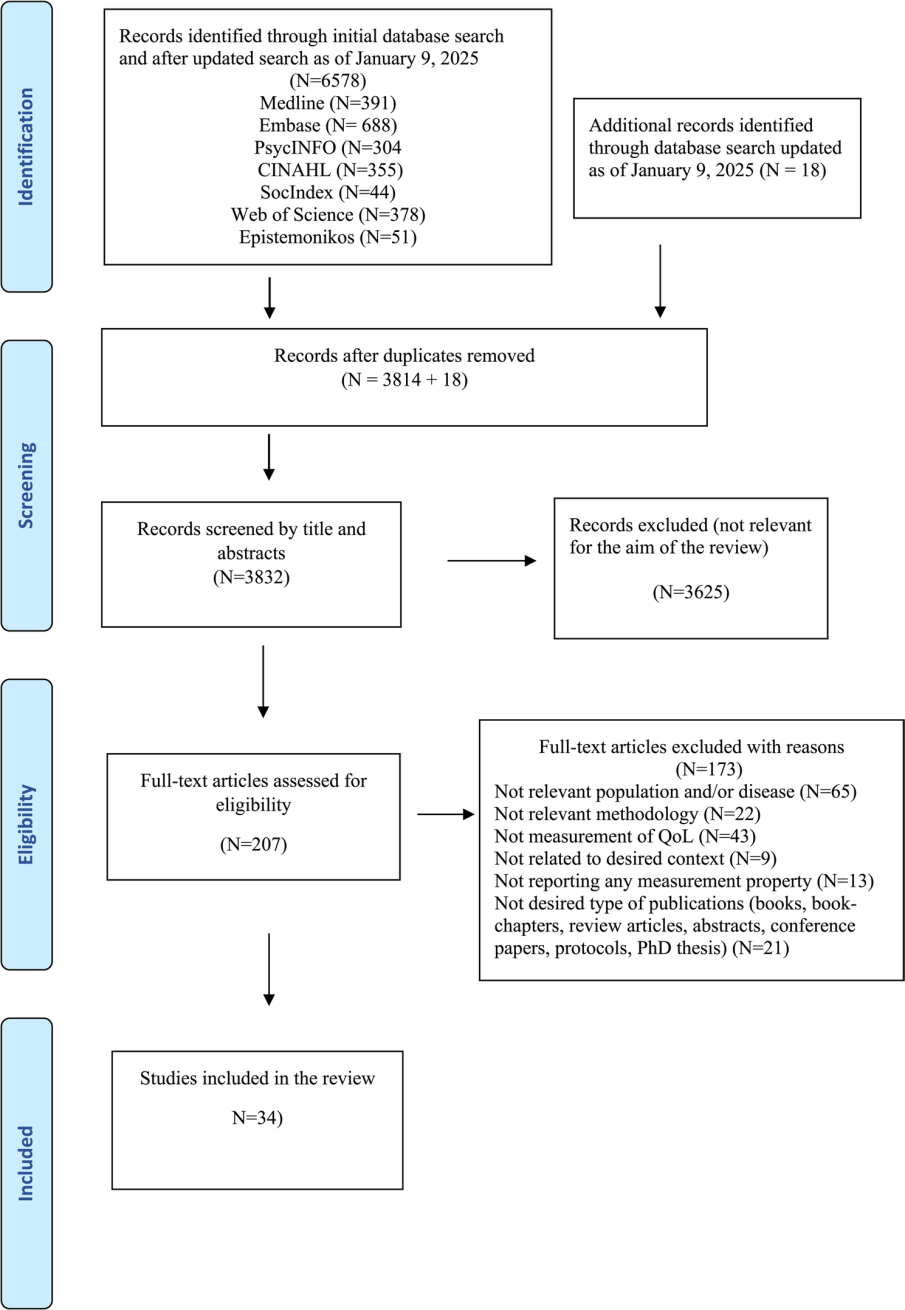
PRISMA Flowchart

A total of 6,578 studies were identified through the initial search conducted in February 2021, and after updating the systematic search in January 2025. Following the removal of duplicates, 3,832 studies were screened based on titles and abstracts, and 207 articles were included for full-text assessment. After thorough evaluation, we ultimately included 34 articles in this review. Information regarding the reasons for exclusions during full-text screening to the final included studies is provided in Fig. 1 (PRISMA Flowchart). Studies selected based on titles and abstracts were moved to the full-text review phase. After reading the full texts, the researchers assessed the relevance and consistency of the evidence concerning the objective of the current scoping review. Discrepancies were discussed among all four researchers until consensus was reached.

### Step 5: extracting data, grouping, summarising, and reporting results

A standardised data extraction template was developed, and each pair of reviewers independently carried out the data extraction. For each included paper, the following information was obtained: author(s), year of publication, title, country where the study took place, aim of the study, design, QoL instrument used, sample size and composition, mean age of caregiver and care recipient, hours spent caring per week and duration of care, relationship between caregiver and care recipient, and whether they cohabit. Table 1 provides a summary of the studies’ characteristics.

**Table 1:** Characteristics of included studies

Author(s), year, title, (country of study) [Ref.nr.] These references occur in the reference list with*	Aim of the study	Design	QoL- Instrument	Sample a. size b. % female	a. Mean age of carer (range) b. Age of care recipient	a. Hours caring per week b. Duration of caring (years)	a. Relationship of carer and care recipient b. Carer living with care recipient
Alzaben et al., (2024). The Psychological Symptoms and Their Relationship to the Quality of Life Among Dementia Patients Caregivers. (Jordan) [57*]	To identify psychological symptoms (depression and anxiety) and their relationship to the quality of life among dementia patients’ caregivers, and whether there are differences in the level of each of them due to the gender variable	Cross-sectional	EQ-5D-5 L	a. *N* = 174 b. 55.17%	a. 20–47 years (M = 31.4) b. Information not provided	a. Information not provided b. Information not provided	a. Information not provided b. Information not provided
Anderson et al. (2022). A Comparative Analysis of Family Quality of Life Between Heterosexual and Sexual Minority Caregivers of People with Dementia (USA) [58*]	To describe the characteristics and family quality of life of SM and heterosexual caregivers for people with Alzheimer’s disease or related dementias (ADRD).	Cross-sectional	FQOLD	a. *N* = 415 b. 53%	a. 45.7 ± 15.8 b. Information not provided	a. Information not provided b. Information not provided	a. Spouse/partner 191 (46%), daughter 86 (21%), son 36 (8%), another relative/friend 102 (25%). b. 210 (51%)
Brown et al. (2019). Measuring the quality of life of family carers of people with dementia: development and validation of C‑DEMQOL (UK) [41*]	To develop a condition-specific measure of the QOL of family carers of people with dementia, applicable across the range of caring situations and severity in dementia.	Mixed methods	C-DEMQOL	a. *N* = 300 b. 72.6%	a. 62 years b. Information not provided	a. Information not provided b. Information not provided	a. 49.4% son/daughter, 42.8% spouse/long term partner, 1.3% family friend, 1.0% sibling, 0.6% other family member, 4.6% other. b. 50.5%
Cheung et al. (2020) Development and evaluation of the Singapore Caregiver Quality of Life Scale - Dementia (Singapore) [59*]	To develop and validate a QOL measurement scale in the English language for caregivers of PWD.	Pilot and Pre-post	SCQOLS-D	a. *N* = 102 b. 80.4%	a. 55 years b. Information not provided	a. 44 h of caring per week. b. Information not provided.	a. 4.9% spouse, 86.3% son or daughter, 8.8% other relatives. 88.2% were ethnic Chinese b. Information not provided.
Daley et al. (2022). Covid-19 and the quality of life of people with dementia and their carers-The TFD-C19 study. (UK) [60*]	To understand how COVID-19 had affected the QoL, wellbeing, and care of people with dementia and their family carers.	Pre-post	C-DEMQOL	a. *N* = 248 b. 68.1%	a. 70.08 years (SD = 10.60) b.77.47 years (SD = 8.03)	a. Information not provided b. Information not provided	a. Spouse/partner 79.4%, son/daughter 19.8%, other 0.8%. b.63.3%
Gumikiriza-Onoria et al. (2024). Psychological distress among family caregivers of persons with Alzheimer’s disease and related dementias in Uganda (Uganda) [61*]	To examine psychological distress, depression, and QoL among family caregivers of patients with Alzheimer’s disease and related dementias in Wakiso District, Uganda.	Cross-sectional	C-DEMQOL	a. *N* = 90 b. 82.2%	a. Females had a median age of 52 years, and males had a median age of 35 years b. Information not provided	a. Information not provided b. Information not provided	a. Information not provided b. Information not provided
Hicks et al. (2022). A cohort study of the impact of COVID-19 on the quality of life of people newly diagnosed with dementia and their family carers (UK) [62*]	To investigate if the impact of the COVID-19 pandemic would be to decrease QoL for people with dementia and their family carers.	Pre-post	C-DEMQOL.	a. *N* = 206 b. 69%	a.66.5 years (SD = 13.86) b. 80.3 years (SD = 8.25)	a. 5.9 h (SD = 6.78) per day caring b. Information not provided	a. Information not provided b. 67%
Horton et al. (2021). Measuring Quality of Life in Carers of People with Dementia: Development and Psychometric Evaluation of Scales measuring the Impact of DEmentia on CARers (SIDECAR) (UK) [63*]	To develop a new instrument to measure the QoL of dementia carers (family/friends)	Longitudinal validation study	SIDECAR	a. *N* = 566 b. 72.3%	a. 70 years b. Information not provided	a. Information not provided b. Information not provided	a. 74.4% spouse/partner, 18.9% son or daughter, 1.9% son-in-law or daughter-in-law, 3.4% other (relative/friend); 97% were of white ethnicity b. 80.9%
Hu et al. (2023). Associations between Affiliate Stigma and Quality of Life among Caregivers of Individuals with Dementia: Mediated Roles of Caregiving Burden and Psychological Distress (Taiwan) [64*]	To test the proposed theoretical model and seek clarification of how affiliated stigma is related to caregiving burden, psychological distress, and QoL.	Cross-sectional	WHOQOL-BREF (Taiwanese version)	a. *N* = 275 b. 52.7%	a. 52.71 years (SD = 12.17) b. 79.21 years (SD = 6.71)	a. Information not provided b. 2.50 years (SD = 2.50)	a. Spouse 37 (13.5%), children 170 (61.7%), others 68 (24.8%). b. 195 (70.9%)
Ibrahim et al. (2024). Burden of care and quality of life among informal caregivers to Alzheimer patients in Egypt (Egypt) [65*]	To evaluate the burden of care and quality of life among informal caregivers of Alzheimer’s patients in Egypt.	Cross-sectional	Short Form Health-Related Quality of Life Translated Scale (HRQOL).	a. *N* = 550 b. 73.5%	a. 43.13 years (± 7.09) b. 67.17 years (± 8.12)	a. <6 h per day 55.5%, ≥ 6 h per day 44.5%. b. <5 years 42%, ≥ 5 years 58%	a. (Spouse, son, daughter…) First degree relative 45.1%, second relatives 37.1%, third relatives 17.8%. b. 440 (80%)
Jeon et al. (2024). Impact of carer burden, social support, and gratitude on quality of life in families of older adults with dementia (South Korea) [66*]	To provide baseline data to inform the development of interventions aimed at improving quality of life, by determining how carer burden, social support, and gratitude affect the quality of life of these families	Cross-sectional	The modified and supplemented version of the Beach Center’s Family Quality of Life in Dementia Scale	a. *N* = 156 b. 80.1%	a. ≤40 years *N* = 51 41–50 years *N* = 61 ≥ 51 years *N* = 44 b. ≤70 years *N* = 32 71–80 years *N* = 79 ≥ 81 years *N* = 45	a. ≤3 h per day *N* = 50 4–7 h per day *N* = 74 ≥ 8 h per day *N* = 32 b. Information not provided	a. Daughter *N* = 76; Son *N* = 20; Daughter-in-law *N* = 33; Son-in-law *N* = 4; Grandchild *N* = 20; Other *N* = 3. b. Information not provided
Kaewwilai et al. (2022). Coping strategies and quality of life among Thai family carers of community-dwelling persons living with dementia: A cross-sectional study (Thailand) [67*]	To examine the association between coping strategies and quality of life (QOL) among Thai family carers of persons living with dementia.	Descriptive correlational quantitative design.	WHOQOL- BREF (Thai version)	a. *N* = 86 b. 87.2%	a. 32–81 years (SD = 9.52). b. 82.10 years (SD = 7.59)	a. 2–24 h per day (SD = 8.47) b. 0.50–20.00 years (SD = 3.96)	a. Wife/Life partner 3 (3.5%), Son/Daughter 74 (86.0%), Son/Daughter-in-law 2 (2.6%), Sister/Brother 3 (3.5%), Sister/Brother-in-law 1 (1.2%), Friend 1 (1.2%), Nephew/Niece 2 (2.3%). b. Information not provided
Kalaitzaki et al. (2022). Dementia Family Carers’ Quality of Life and Their Perceptions About Care-receivers’ Dementia Symptoms: The Role of Resilience (Greece) [68*]	To examine (a) the relationship between caregivers’ (CG) quality of life (QoL) and their reports about care receivers’ dementia symptoms and (b) whether CG’s resilience would be a mediator in this relationship.	Cross-sectional	WHOQOL-BREF (Greek version)	a. *N* = 118 b. 78.8%	a. 58.9 years (SD = 11.5) b. 82.7 years (SD = 7.4)	a. 11.8 h per day (SD = 8.8) b. 60.2 months – an average of 5 years	a. Children 62.7%, Spouses 25.4%, Neighbour/other 11.9%
Kimura et al. (2021). Young- and Late-Onset Dementia: A Comparative Study of Quality of Life, Burden, and Depressive Symptoms in Caregivers (Brazil) [69*]	To compare the quality of life, subjective burden, and depressive symptoms of caregivers in young onset dementia and late onset dementia and to further investigate which factors might be associated with QoL, burden, and depressive symptoms in both groups of caregivers.	Cross-sectional	QoL-AD	a. *N* = 110 b. 77.3%	a. 54.70 years (SD = 14.5) b. 71.69 years (SD = 9.5)	a. Information not provided b. 45.68 months (SD = 37.2)	a. Wives/husbands 40.0%, daughters/sons 45.5%, others 14.5%.
Kim & Cha (2022). Effect of Perceived Stress on Health-Related Quality of Life among Primary Caregiving Spouses of Patients with Severe Dementia: The Mediating Role of Depression and Sleep Quality (South Korea) [70*]	To examine the mediating effects of depression and sleep quality on the impact of perceived stress on the HRQoL of the primary caregiver spouses of patients with severe dementia.	Cross-sectional	EQ-5D (Korean version)	a. *N* = 229 b. 62.5%	a. 10.4% were under 65 years old, 28.0% were 65–74 years old, 52.0% were 75–84 years old, and 9.6% were 85 years old or older. b. Information not provided.	a. Information not provided b. Information not provided	a. Information not provided b. Information not provided
Lee et al. (2021). Development of a short form of the Singapore Caregiver Quality of Life Scale – Dementia: SCQOLS-D-15 (Singapore) [71*]	To develop a short form of the SCQOLS-D and evaluate its measurement properties	Test-retest	SCQOLS-D-15	a. *N* = 102 b. 80.4%	a. 54.6 years (SD = 10.6), (22–86); 88.2% ethnic Chinese. b. Information not provided	a. Average of 43.5 h per week b. Information not provided	a. 86.3% son/daughter b. Information not provided
Liu et al. (2021). Finding a balance in family caregiving for people with dementia: A correlational longitudinal study (Taiwan) [72*]	This study aimed to (1) identify different balance trajectories for family caregivers of people with dementia; (2) explore predictors of trajectory group membership, and (3) examine associations of different balance trajectories with caregiving outcomes, i.e., depressive symptoms and health-related quality of life.	Correlational longitudinal design	SF- 36	a. *N* = 200 b. 53.5%	a. 54.7 years (SD = 10.5) b. 81.0 years (SD = 7.0)	a. 8.1 h/day (SD 7.5) b. 67.8 months (SD 64.6)	a. Spouse 34%, son 74%, daughter 53%, daughter-in-law 36%, other 3%. b. *N* = 154 (77%)
Lucijanić et al. (2021). Predictors of Health-related Quality of Life in informal caregivers of dementia patients in Zagreb, Croatia, a cross-sectional study (Croatia) [73*]	To estimate health-related quality of life (HRQoL) in family-member caregivers of patients with dementia in Croatia and to assess relevant predictors.	Cross-sectional	SF-36	a. *N* = 131 b. 67.9%	a. 62.1 *±* 13. b. 79.4 *±* 7.1	a. Information not provided b. 2 years (1–3)	a. Spouse *N* = 51 (38.9%), children *N* = 67 (51.1%), other *N* = 13 (9.9%) b. Information not provided
Meichsner et al. (2019). Telephone-Based Cognitive Behavioral Intervention Improves Dementia Caregivers’ Quality of Life (Germany) [74*]	To examine the effects of a telephone-based cognitive behavioural intervention on dementia caregivers’ quality of life (QoL)	RCT	WHOQOL-BREF (German version)	a. *N* = 273 b. 80.6%.	a. 64.10 years (SD = 11.04) range: 23–91 b. 78.76 years (SD = 9.35, range: 44–104 years)	a. Information not provided b. 4.89 years (SD = 3.68, range: 5 months-19 years),	a. Spouse (*N* = 165, 60.4%), child (*N* = 104, 38.1%). b. *N* = 218 (79.9%)
Magteppong et al. (2021). The Effects of the Modified Transtheoretical Theory of Stress and Coping (TTSC) Program on Dementia Caregivers’ Knowledge, Burden, and Quality of Life (Thailand) [75*]	To examine the effect of the modified transtheoretical theory of stress and coping (TTSC) program on the knowledge, burden, and quality of life of dementia caregivers.	Quasi-experimental	WHOQOL-BREF (Thai version)	a. *N* = 62 b. 74.2% experimental group and 87.1% in the control group	a. 41–60 years; 53.06 years for the experimental group and 52.52 years for the control group b. Information not provided	a.6 – more than 12 h per week b. 0 – more than 9 years	a. In experimental group: Spouse 16.1%, son (blood relatives) 19.4%, daughter (blood relatives) 48.4%, Relative 9.7%, Friend/adopted child/Neighbour 6.5% In control group: Spouse 16.1%, son (blood relatives) 9.7%, daughter (blood relatives)58.1%, relative 6.5%, friend, Adopted child/neighbour 9.7%. b. Information not provided.
McKenna et al. (2020). International Development of the Alzheimer’s Patient Partners Life Impact Questionnaire (APPLIQue) (UK) [76*]	To develop a new outcome measure to assess AD caregivers’ quality of life (QoL).	Mixed-methods	APPLIQue	a. *N* = 116 b. 75.9%	a.70.3 years (SD = 9.6) b. Information not provided.	b. Mean 6.6 (SD = 9.0) years	a. 116 spouse b. 100%
Monteiro et al. (2024). Burden and quality of life of family caregivers of Alzheimer’s disease patients: the role of forgiveness as a coping strategy (Portugal) [77*]	To evaluate the variables associated with, contributing to, and moderating QoL and the burden on family caregivers.	Cross-sectional	SF-36	a. *N* = 130 b. 77.6%	a. 66.15 years (SD = 13.64) b. 3.98 years (SD = 2.20)	a. 5.38% 0–12 h per day. 94.6% 13–24 h per day b. 0.5-9 years (SD = 2.2)	a. Children (SD = 42,3), companion (SD = 44.6), others (SD = 13.1) b. Information not provided
Morrison et al. (2020). The Impact of Risk and Resistance Factors on Quality of Life in Caregivers of Individuals with Dementia (USA) [78*]	To investigate the effect of caregiver and care recipient risk and resistance factors on caregiver quality of life (QOL).	Cross-sectional	CQOL-ND	a. *N* = 103 b. 68%	a. 67.45 years (SD = 12.56) b. Information not provided	a. Information not provided b. Information not provided.	a. Partner/Spouse 64 (62.1%), Child 28 (27.2%), Other 11 (10.7%) b. Information not provided
Nasreen et al. (2024). Caregiver burden, mental health, quality of life and self-efficacy of family caregivers of persons with dementia in Malaysia: baseline results of a psychoeducational intervention study (Malaysia) [79*]	To examine the determinants of outcome measures (caregiver burden, depressive and anxiety symptoms, quality of life and caregiving self-efficacy) among Malaysian family caregivers to persons with dementia.	Cross-sectional	CASP-19	a. *N* = 121 b. 69.4%	a.51.6 years (SD = 12.7) b. 75.2 years (SD = 10.1)	a. 18.6 h per day (SD = 6.9) b. 47.9 months (≅ 4 years) (SD = 42.8)	a. Spouse = 27.3% Adult child = 62.8% In-laws = 9.9% b. Information not provided.
Oliveira et al. (2018). The development and validation of the Dementia Quality of Life Scale for Older Family Carers (DQoL-OC) (UK) [80*]	To develop and validate a unique age and dementia-specific QoL scale for use with older family carers of people with dementia – the Dementia Quality of Life Scale for Older Family Carers (DQoL-OC) presents the process of item reduction and the psychometric evaluation of the final scale version.	Sequential exploratory mixed-methods design	DQoL-OC	a. *N* = 182 b. 65%	a. 72.2 b. Information not provided	a. 55% 12 h a day b. 78% 1–6 years	a.80% spouses b.80%
Pereira et al. (2021). Contributors and Moderators of Quality of Life in Caregivers of Alzheimer´s Disease Patients (Portugal) [81*]	To identify the variables that contributed to Quality of Life (QoL) of Alzheimer’s Disease (AD) caregivers, considering the caregiving context, stressors, role strains, and resources.	Cross-sectional	QoL-AD	a. *N* = 102 b. 79.4%	a. 53.14 years (SD *=* 13.33). b. 54–93 years (SD = 8.67)	a. 1–12 h/day (39.2%) 13–24 h/day (60.8%) b. 0–25 years (SD = 4.16)	a. Information not provided b. Information not provided
Pothiban et al. (2020). Quality of life and the associated factors among family caregivers of older people with dementia in Thailand (Thailand) [82*]	To describe the level of QOL among dementia family caregivers, and to explore factors associated with predicting the QOL among dementia family caregivers living in the communities in Thailand.	Cross-sectional	EQ-5D-3 L	a. *N* = 76 b. 77.63%	a. age < 60 years (78.94%) with age ranging from 32 to 77 years and a mean of 52.13 ± 10.23 years, b. Information not provided	a. Information not provided b. Information not provided	a. Information not provided b. Information not provided
Potter et al. (2023). Living well while providing support: validation of LTCQ‑Carer for assessing informal carers’ quality of life (UK) [83*]	To understand if LTCQ-Carer provided a valid means of assessing care-related quality of life, and if use of LTCQ-Carer could fit into an existing clinical pathway, such that carers’ needs could be identified and supported concurrently with patients’ needs.	Mixed methods	LTCQ	a. *N* = 107 b. 63%	a. Information not provided. The age of the family caregivers ranged between 41 and 90 years. b. Information not provided.	a. Information not provided b. Information not provided	a. Information not provided b. 81%
Read et al. (2024). Long-term impact of the COVID-19 pandemic on the quality of life of people with dementia and their family carers (UK) [84*]	To examine QoL trajectories of a cohort of people newly diagnosed with dementia months before the first coronavirus disease 2019 (COVID-19) outbreak and investigate how these trajectories varied using multiple follow-ups and the different dimensions of QoL.	Longitudinal observational design.	C-DEMQOL	a. *N* = 206 b. 69%	a.66.5 years (SD = 13.86) b. 80.3 years (SD = 8.25)	a. 5.9 h (SD = 6.78) per day caring b. Information not provided	a. Information not provided b. 67%
Sittironnarit et al. (2020). Quality of life and subjective burden of primary dementia caregivers in Bangkok, Thailand (Thailand) [85*]	To study and compare factors correlated with quality of life and subjective burden of primary dementia caregivers at the Psychiatric Outpatient Unit of Siriraj Hospital in Bangkok, Thailand.	Cross-sectional	PTQL	a. *N* = 155 b. 80%	a.52.2 years (+ 13.3) b. 79.2 years (± 8.5)	a. 11.6 h per day (± 8.7) b.5.9 years (± 4.6)	a. Children 63.2%, Spouse 13.5%, Formal caregiver 2.6%, Other 20.6% b. 81.3%
Silarova et al. (2023). Feasibility, validity and reliability of the ASCOT-Proxy and ASCOT-Carer among unpaid carers of people living with dementia in England (UK) [86*]	To establish the feasibility, construct validity and reliability of the ASCOT-Proxy and ASCOT-Carer, with unpaid carers of people with dementia living at home unable to self-report, and to establish structural characteristics of the ASCOT-Proxy.	Cross-sectional	ACOT-Carer	a. *N* = 313 b. 75.72%	a. 62.44 years (SD = 12.04) b. 81.47 years (9.37)	a. 50 h or more 46.96% 49 h or less 52.08% b. Information not provided	a. Husband/wife/partner 41.53%, parent (mother, father) 48.88%, Sibling 1.28%, Other 8.31% b. 57.83%
Uyar et al. (2019). Assessment og the impact of dementia care and support program in both patient and caregiver outcomes: an intervention study (Turkey) [87*]	To provide evidence for the impact of multicomponent and multidisciplinary interventions on patient and caregiver outcomes.	RCT	SF-36	a. *N* = 61 b. 78.6%	a. 22–81 years (SD = 53.6 ± 14.8) b. ≤76 years (34.2%) > 76 years (63.9%)	a. Information not provided b. Information not provided	a. Spouse (26.2%), child (49.1%), daughter-in-law (18.0%), sibling (1.0%).
Wang et al. (2020). Job Demands and the Effects on Quality of Life of Employed Family Caregivers of Older Adults With Dementia: A Cross-Sectional Study (Taiwan) [88*]	To examine how job demands influenced the quality of life for employed family caregivers of older adults with dementia in Taiwan.	Cross-sectional	SF-36	a. *N* = 214 b. 46.7%	a. Information not provided (age ≥ 18 years to be included in the study) b. Mean age of 78.66 years	a. Information not provided b. Information not provided	a. Son (52.3%), daughter (24.8%), daughter-in-law (22.0%), son-in-law (0.9%). b. Information not provided
Yazdanmanesh et al. (2023). Relieving care burden and promoting health-related quality of life for family caregivers of elderly people with Alzheimer’s disease via an empowerment program (Iran) [89*]	To determine the impact of the empowerment program on the care burden and HRQoL of family caregivers of elderly people with Alzheimer’s disease.	RCT	SF-12	a. *N* = 72 b. 73.6%	a. 47.4 ± 5.9 in intervention group and 45.7 ± 5.7 in control group b.74.8 ± 5.8 years in the intervention group and 73.6 ± 2.6 years in the control group	a. Information not provided b. Information not provided	a. Spouse 13.8%, child 59.6%, brother/sister 5.5%, others 22.2% b. Information not provided.

Similarly, an extraction template was created, including all QoL instruments described in each study. The template recorded the instrument’s name, its construct, number of domains and items, response options, development process, language(s), mode of administration, duration, validity, and reliability.

To present the information on the measurement properties of the instrument used to assess QoL in informal caregivers of people with dementia, all reported validity and reliability measures, including Cronbach’s alpha and/or McDonald’s omega [90], were documented in the extraction template based on the sample investigated. Like Cronbach’s alpha, McDonald’s omega measures internal consistency; however, it is often regarded as more accurate and robust because it considers the multidimensionality of the data [91]. Missing details about the studies’ characteristics or the instruments were marked as “Information not provided”.

As suggested by Pollock et al. [91], the data relevant to the research question of the scoping review were extracted. Relevant data are summarised and presented in two tables (Tables 1 and 2).

**Table 2:** Characteristics of QoL instruments

QoL instrument (authors, year)				Construct	i. Number of domains ii. Items iii. Response categories	i. Construct/Instrument Development* ii. Language(s)	Administration i. mode ii. time	Measurement proprieties	
								Reliability	Validity
APPLIQue (McKenna et al. 2020) [76*]				APPLIQue (The Alzheimer’s Disease Pat) is a questionnaire specific to spousal caregivers of people with AD. It is based on a clear conceptual model: the need-based model of QoL.	i. Six domains: Energy level, Pain, Emotional reactions, Sleep, Social isolation, Physical mobility ii.25 iii. 0–25	i. The questionnaire’s content was derived from unstructured qualitative interviews conducted with caregivers in the UK, Germany, Italy, Spain, and the US. ii. English, German, Italian, Spanish	i. Individual interviews and postal survey ii. Information not provided	Internal Consistency: Cronbach’s alpha *≥* 0.93 Test-Retest Reliability: Spearman correlation coefficient = 0.88	Content validity is reasonable, based on qualitative interviews with caregivers to ensure they accurately reflect the caregiving experience. Face validity: adequate, interviews supported the face validity of the tool​
ASCOT Carer (Silarova et al., 2023) [86*]				ASCOT-Carer (The Adult Social Care Outcomes Toolkit—ASCOT) is a version of the ASCOT Proxy questionnaire that examines aspects of life that are important to friends and relatives who care for someone with social care support needs.	i. Seven domains: Occupation, Control over daily life, Self-care, Personal safety, Social participation, Space and time to be yourself, Feeling supported and encouraged. ii. Seven iii. 0–21	i. The Adult Social Care Outcomes Toolkit (ASCOT) is a suite of self-report, interview, easy-read, or mixed-methods measures designed to measure social-care-related quality of life (SCRQoL) of service users and their carers. An adapted version of the ASCOT self-completion version (SCT4), the ASCOT-Proxy has been developed for completion by someone who knows the person well, such as, a close friend or relative. ii. English	i. Self-administered questionnaire ii. Information not provided.	Internal Consistency is good, as indicated by Zumbo’s ordinal alpha = 0.70. Rasch analysis assessed the scale’s structural validity, ensuring that the items accurately measured the intended dimensions of SCRQoL.	Construct validity: good. It confirms that it measures the concept it was designed to assess – social care-related quality of life for carers (SCRQoL).
CASP-19 (Nasreen et al., 2024) [79*]				Control, Autonomy, Self-Realization, and Pleasure (CASP-19) is an age-specific quality of life measure.	i. Four domains: control, autonomy, self-realisation and pleasure ii. 19 items iii. Each item is scored on a four-point scale (‘Often’, ‘Sometimes’, ‘Not often’ and ‘Never’). Total scores range from 0 to 57. The higher the score, the better the perceived QoL	i. CASP-19 helps measure QoL in older people as it can measure QoL directly and can stand alone as a summative index ii. Malay	i. Individual telephone interviews. ii. Information not provided.	CASP-19 showed good reliability in the study with a Cronbach’s alpha of 0.88.	Information not provided.
C-DEMQOL	Brown et al. (2019) [41*]			The Caregiver Quality of Life (C-DEMQOL) instrument was designed to ensure comprehensive coverage of relevant domains while prioritising psychometric efficiency and usability in research practice. It is a condition-specific measure of the QOL of family carers of people with dementia, applicable across the range of caring situations and severity in dementia.	i. Five domains: Carer-patient relationship, Carer wellbeing, Meeting personal needs, Confidence in the future, Feeling supported ii. 30 items iii. 5 − 1	i. Instrument developed from The Caregiver Quality of Life (CGQOL) instrument, an extended 80-item inventory, assesses 10 domains of carer experiences, including the objective characteristics of the caregiving situation (for example, whether the carer assisted with specific activities) in addition to the more direct indicators of QOL such as worry. ii. English	i. Interviews ii. 6–60 min. 50% complete in 15 min	Cronbach’s alpha of the total C-DEMQOL scales ranged from 0.80 to 0.93. The average convergent correlation is 0.58, and the average discriminant is 0.40. Factor analysis confirmed that C-DEMQOL sum scores are reliable in measuring overall QOL (*ω* = 0.97) and its five subdomains: ‘meeting personal needs’ (*ω* = 0.95); ‘carer wellbeing’ (*ω* = 0.91); ‘carer-patient relationship’ (*ω* = 0.82); ‘confidence in the future’ (*ω* = 0.90) and ‘feeling supported’ (*ω* = 0.85).	The factorial structure was evaluated based on polychoric correlations of ordinal item responses (Grade Response Model), favouring a five-factor solution.
	Daley et al. (2022) [60*]			A measure of QoL in family carers of people with dementia. A 30-item measure with scores ranging from 30–150 with higher scores representing better QoL for family carers. The measure provides a rating for overall QoL, which is made up from five subscales: meeting personal needs, carer wellbeing, carer-patient relationship, confidence in the future, and feeling supported.	i. Five domains: Carer-patient relationship, Carer wellbeing, Meeting personal needs, Confidence in the future, Feeling supported ii. 30 items iii. 5 − 1	i. Information not provided; however, the authors refer to Brown et al. (2019) ii. Information not provided, but the study was conducted in the UK	i. Interviews were conducted by telephone ii. Information not provided.	The total QOL score was reliable, with omega (ω = 0.97), ranging from 0.82 to 0.95 for each of the five sub-domains.	Information not provided.
	Gumikiriza-Onoria et al., (2024) [61*]			The Caregiver Dementia Quality of Life Measurement was used to evaluate caregiver quality of life in combination with other instruments, thus facilitating a detailed analysis of the psychological and situational aspects of caregivers’ experiences.	i. Five domains: Carer-patient relationship, Carer wellbeing, Meeting personal needs, Confidence in the future, Feeling supported ii. 30 items iii. 5 − 1	i. Information not provided ii. English or Luganda	i. Interviews ii. 90–120 min.	Internal consistency, measured with Cronbach’s alpha, was 0.87.	Information not provided.
	Hicks et al., (2022) [62*]			The Caregiver Dementia Quality of Life Measurement (range 30–150) to assess carer QoL. This interviewer-administered, dementia-specific questionnaire assesses how individuals have felt in their caregiving role over the past four weeks.	i. Five domains: Carer-patient relationship, Carer wellbeing, Meeting personal needs, Confidence in the future, Feeling supported ii. 30 items iii. 5 − 1	i. Information not provided; however, the authors refer to Brown et al. (2019) ii. Information not provided, but the study was conducted in the UK	i. Interviews conducted by telephone ii. Information not provided	Cronbach’s alpha of 0.92. Initial level (intercept) of QoL (C-DEMQOL) was 100.4 (standard errors [SE] = 1.3, *P* < 0.001	Information not provided
	Read et al. (2024) [84*]			The 30-item C-DEMQOL (range 30–150) was used to assess care QoL. It is an interview-administered, dementia-care-specific questionnaire that assesses QoL over the past four weeks.	i. Five domains: Carer-patient relationship, Carer wellbeing, Meeting personal needs, Confidence in the future, Feeling supported ii. 30 items iii. 5 − 1	i. Information not provided; however, the authors refer to Brown et al. (2019) ii. Information not provided, but the study was conducted in the UK.	i. Interviews conducted by telephone ii. Information not provided	Cronbach’s alpha of 0.90 (or higher for all time points) QoL subscales 0.72–0.94	Information not provided
CQOL-ND (Morrison et al., 2020) [78*]				The Caregiver Quality of Life Index – Neurocognitive Disorder (CQOL-ND) scale was used to measure caregiver QOL, the study’s primary outcome. The scale measures the impact of the care recipient’s illness on family caregivers in various life areas.	i. Six domains including physical, emotional, family, social, spiritual, and eco-nomic functioning (e.g., “My sleep is less restful,” “My economic future is uncertain,” “My sense of spirituality has increased,” “I am satisfied with the support I get from my family”) ii. 35 items iii. Five-point Likert-type scale ranging from 0 (not at all) to 4 (very much)	i. The CQOL-ND was adapted for this study from the Caregiver Quality of Life Index – Cancer (CQOLC)scale ii. English	i. Self-reported questionnaires can be completed online or on paper in the clinic. ii. Information not provided.	For this study, the CQOL-ND had excellent reliability (Cronbach’s alpha = 0.898). Test-retest reliability was 0.95, and internal consistency was 0.91	Information not provided.
DQOL-OC (Oliveira et al., 2017) [80*]				The Dementia Quality of Life Scale for Older Family Carers has a 22-item structure represents various QoL domains which are relevant to older carers, such as social relationships, financial situation; psychological health; independence; control over life event & freedom; leisure; social and solo activities; physical health; general health; energy and vitality; satisfaction with life and caregiving; identity; and life in general.	i.10 domains ii. 22 items iii. 1–5	i The instrument development took place in two steps, and this was the second step in reducing the first 100 items developed through qualitative focus groups. ii. English	i. Self-reported questionnaires. ii. Information not provided	DQOL-OC 22-item positively correlated with other scales’ scores: WHOQOL-AGE; SWLS; PHS-VAS; OHRQOL-VAS. Cronbach’s alpha of 0.94. Test–retest study: lower bound *r* = 0.835; *p* < 0.001	Information not provided.
EQ-5D (Kim & Cha, 2022) [70*]				The European Quality of Life Five Dimension (EQ-5D) scale intends to measure the extent to which an individual is currently experiencing problems in mobility, self-care, usual activities, pain/discomfort, and anxiety/depression. The EQ-5D index ranged from − 0.171 to 1, with higher indexes indicating higher quality of life	i. Number of domains: 5 ii. Information not provided iii. Response categories: three-response Likert scale with options ranging from 1 (none) to 3 (severe problem).	i. Information not provided ii. Korean	(i) and (ii) Information not provided.	The study showed that Cronbach’s alpha was 0.79	Information not provided.
EQ-5D-3 L		Pothiban et al. (2020) [82*]		The EuroQoL Five Dimensions Questionnaire (EQ-5D-3 L) Thai Version. Tongsiri (2009) translated this questionnaire into Thai. The EuroQol Office team granted permission to use this instrument.	i. The scale consists of five dimensions. ii. Five items measuring five dimensions: mobility, self-care, usual activities, pain/discomfort and anxiety/depression; and the Visual Analogue Scale (VAS). All items are converted to the utility score by applying a formula that attaches values to each dimension’s levels, ranging from − 0.454 to 1.000. iii. Three-option response format of levels of severity (“no problems”, “some problems”, and “severe problems”).	i. In this study, the researchers used only the index part of EQ-5D-3 L to measure the five dimensions. ii. Thai	i. Self-reported questionnaires. ii. Between 45–60 min.	Cronbach’s alpha was 0.92.	Information not provided.
EQ-5D-5 L		Alzaben et al. (2024) [57*]		The EQ-5D-5 L Questionnaire is a validated generic, non-disease-specific instrument for evaluating HRQoL. Aburuz et al. (2009) translated and validated it into Arabic.	i. The scale consists of two parts. The first part of EQ-5D-5 L covers five dimensions of health: mobility, self-care, usual activities, pain/discomfort and anxiety/depression. The second part comprises a Visual Analogue Scale (EQ-VAS) for valuing health states on a rating scale from 0 to 100, with higher scale values indicating better quality of life.	i. The inventory is comprised of 29 items and three dimensions (psychological, social, physical) ii. a four-point scale, from 1 (‘Strongly Disagree’) to 4 (‘Strongly Agree’). ii. Information not provided, but the study was conducted in Jordan.	(i) and (ii) Information not provided.	Cronbach’s alpha ranged between 0.78 and 0.92.	The discriminant validity ranged between 0.31 and 0.89. These figures pertain to construct validity and do not offer direct evidence of content validity, which requires assessment of item relevance, comprehensiveness, and clarity from the perspective of informal caregivers.
FQOL Scale (Jeon et al. 2024) [66*]				The Beach Center Family Quality of Life Scale (FQOL Scale) assesses families’ perceptions of their satisfaction with different aspects of family quality of life.	i. Five domains: Family Interaction, Parenting, Emotional Well-being, Physical/Material Well-being, and Disability-Related Support. ii. 26 items iii. Five-point Likert scale, where 1 = very dissatisfied, 3 = neither satisfied nor dissatisfied, and 5 = very satisfied.	i. Family caregivers’ quality of life was evaluated using a modified and supplemented version of the FQOL Scale. ii. Information not provided, but the study was conducted in South Korea.	i. Self-reported questionnaire ii. Information not provided.	Cronbach’s alpha was 0.94.	Information not provided.
FQOLD (Anderson et al., 2022) [58*]				The Family Quality of Life in Dementia Scale (FQOLD) is a scale designed to assess family quality of life in families caring for someone with Alzheimer’s disease or related dementias (ADRD)	i. Four domains: caregiver support (“My family has outside help available to us to take care of the special needs of all family members.”), disease-related support/medical care (e.g., “My family gets medical care when needed.”), family interactions (e.g., “My family has the support needed to manage our feelings.”), and well-being (e.g., “My family member with dementia has support to thrive in: his/her environment.”). ii. 41-item iii. Response categories: items are rated on a four-point scale, generating a total score, with higher scores indicating higher levels of family QoL	i. Information not provided ii. Information not provided, but the study was conducted in the USA.	(i) and (ii) Information not provided	Cronbach’s alpha was 0.96.	Information not provided.
HRQOL (Ibrahim et al., 2024) [65*]				Health-Related Quality of Life Scale (HRQOL) was used to evaluate the burden of care and quality of life among informal caregivers to Alzheimer patients in Egypt and to develop Short-Form Health-Related Quality of Life Translated Scale (HRQOL). HRQOL scale included multi-item scales to measure eight aspects of health: physical functioning (items 1, 2), bodily pain (items 7, 8), role limitations brought on by physical health issues (items 3, 4), limitations brought on by emotional problems (item 6), emotional well-being (item 5), social functioning (item 10), energy/fatigue (item 9), and general health perceptions (item 11).	i. Eight domains ii.67 items ii. Information not provided	i. Short Form based on the Health-Related Quality of Life Scale (HRQOL) ii. Information not provided, but the study was conducted in Egypt.	i. Self-reported questionnaire. ii. 30–45 min.	Cronbach’s alpha was 0.82.	Information not provided.
LTCQ-Carer (Potter et al., 2023) [83*]				Long-Term Conditions Questionnaire for Carers (LTCQ) is a self-administered questionnaire designed to capture a holistic construct of ‘living well with LTCs’. LTCQ-Carer is intended to measure care-related QoL regarding the broader health and social-care related domains captured by LTCQ, which are affected by one or more LTC in the cared-for person.	i. Seven domains taken from LTCQ: Sense of control, ability to do meaningful activities, safety inside and outside the home, burden of treatments and services, negative experiences including loneliness and stigma, confidence to self-manage LTCs, and ability to live as one wants. ii. 21 iii. Five-point Likert scale	LTCQ-Carer was developed through cognitive interviews on draft questionnaire content with carers of people living with mild cognitive impairment (MCI) or dementia. Original phrasing of LTCQ items was retained and adapted to LTCQ-Carer to make more sense from a carer’s perspective. The development of the instrument was done in two phases. ii. Information not provided, but the study was conducted in the UK.	i. Self-reported questionnaire. ii. Information not provided	Internal reliability: Cronbach’s alpha = 0.95. Predicted associations with EQ-5D and ASCOT-Carer are a valid means of capturing quality of life outcomes for informal caregivers supporting people recently diagnosed with MCI or dementia.	Content validity was evaluated through cognitive interviews with informal caregivers. Participants reviewed the draft items of the LTCQ-Carer to ensure they were relevant, comprehensive, and understandable. Construct validity was assessed by examining the associations between LTCQ-Carer scores and scores from existing validated measures, such as the EQ-5D and ASCOT-Carer.
PTQL (Sittironnarit et al., 2020) [85*]				The Pictorial Thai Quality of Life (PTQL) and the Thai version of the Zarit Burden Interview (ZBI) were used to collect general information, quality of life and subjective burden data of primary dementia caregivers.	i. Six domains: physical, cognitive, affective, social function, economic and self-esteem domains. Each domain can be classified as having a good, moderate, or poor quality of life. ii. 25 items iii. Information not provided	i. Information not provided, but the authors refer to (Phattharayuttawat et al., 2005) ii. Thai	i. Self-reported questionnaire ii. Information not provided.	Cronbach’s alpha was 0.88, and values of the six scales ranged from 0.81 to 0.91.	Instrument validity was tested using the WHOQOL-BREF. The Pearson’s R correlation coefficient was 0.92, and the Area under the ROC curve was 0.97.
QOL-AD; The quality of life in Alzheimer disease (QoL AD), Caregiver’s QoL version (C-QoL)			Kimura et al. (2021) [69*]	Assessment of caregiver Quality of life	i. Number of domains: 13 ii. 13 items iii. Response categories: poor (1), fair (2), good (3), or excellent (4). The total score ranges from 13 to 52.	i. Information not provided ii. Information not provided, but the study was conducted in Brazil.	i. Self-reported questionnaire ii. Information not provided	Cronbach’s alpha was 0.86.	Information not provided.
			Pereira et al. (2021) [81*]	Assessment of caregiver Quality of life	(i) Information not provided (ii) 13 items iii. Information not provided	i. Information not provided ii. Information not provided, but the study was conducted in Portugal	(i) and (ii) Information not provided	Cronbach’s alpha was 0.86.	Information not provided.
SCQOL-D-15 (Lee et al., 2021) [71*]				Short-Form of the Singapore Caregiver Quality of Life scale – Dementia (SCQOLS-D-15) is a short form of the instrument developed from SCQOLS-D for rapid evaluation of QoL of Asian family caregivers.	i. Five domains: Physical Well-being, Mental Well-being, Experience & Meaning, Impact on Daily Life and Financial Well-being ii. 15 items iii. Five-point Likert scale ranged from not at all (0) to very much (4)	i. Instrument developed from SCQOLS-D in three steps and added some items from their pilot study. ii. English	i. Self-reported questionnaire. ii. Information not provided	Cronbach’s alpha ranged between 0.76 and 0.90; test-retest reliability was ICC: 0.72–0.93.	Content validity: good based on involving experts and stakeholders in the item selection process.
SCQOLS-D (Cheung et al. 2020) [59*]				The Caregiver-targeted Quality of Life Measure (CGQOL) for PWD caregivers encompasses general themes initially developed with advanced cancer patients in mind. It has been extended to cover dementia specific concerns.	i. Six domains: Physical Well-being, Mental Well-being, Experience & Meaning, Impact on Daily Life, Financial Well-being and one total. ii. 63 iii. Five-point Likert scale.	i. Six investigators individually reviewed each item in the QoL measures ACQLI, PIXEL, and CGQOL in a pilot study. An additional three items were included. ii. English	i. Self-reported questionnaire ii. Information not provided	Cronbach’s alpha of the total and domain scores ranged from 0.89 to 0.95 Test-retest reliability (intraclass correlation coefficient) ranged from 0.77 to 0.92	Content validity is good, as it involves caregivers and healthcare professionals during development. The items were generated through literature review, expert consultations, and focus group discussions, ensuring the relevance and representativeness of the scale’s domains. Feedback from stakeholders was used to refine the items.
SF-12	Yazdanmanesh et al. (2023) [89*]			The 12-item Short Form Health Survey (SF-12) was used to measure HRQoL. The SF-12 comprises 12 questions and measures HRQoL in two subscales: mental and physical.	i. Two domains used in the study ii. 12 questions iii. Information not provided other than this minimum score obtained from this survey is 12, and the maximum is 48. The higher the score, the higher HRQoL	i. Information not provided ii. Persian	i. Self-reported questionnaire ii. Information not provided	Cronbach’s alpha ranged between 0.89 and 0.90.	Information not provided.
SF-36	Liu et al. (2021) [72*]			Health-related quality of life, which includes physical and mental health outcomes, was measured using the Physical Health Component Summary and Mental Health Component Summary of the Taiwan-version Medical Outcomes Study SF-36, which was demonstrated to have good psychometric properties (Tseng et al., 2003)	i. Eight health domains: physical functioning, role disability due to physical health problems, bodily pain, vitality, general health perceptions, social functioning, role disability due to emotional issues, and general mental health. ii. 36 items covering the eight health domains) iii. Information not provided, except for algebraic sums, was computed for each concept. The raw scale scores are transformed to a 0-100-point scale, with higher scores representing better health-related quality of life	i. Information not provided ii. Taiwanese	i. Self-reported questionnaire ii. Information not provided	Cronbach’s alpha ranged between 0.86 and 0.95.	Information not provided.
	Lucijanić et al. (2021) [73*]			The 36-Item Short Form Health Survey (SF-36) was used to investigate HRQoL in family members of patients with dementia who are informal caregivers and to assess relevant HRQoL predictors in the Croatian population.	i. Eight domains: psychical functioning (PF), role physical (RP), bodily pain (BP), general health (GH), vitality (VT), social functioning (SF), role emotional (RE) and mental health (MH), ii. 36 items iii. Results are provided with a score of 0-100 points. A higher score represents better HRQoL.	i. Information not provided ii. Information not provided, but the study was conducted in Croatia.	(i) and (ii) Information not provided.	Cronbach’s alpha ranged between 0.93 and 0.76 for all subscales.	Information not provided.
	Monteiro et al. (2024) [77*]			Together with other instruments, the Short Form Health Survey (SF-36) was used to evaluate the variables associated with, contributing to, and moderating the quality of life (QoL) and burden in family caregivers.	i. Two dimensions of QoL (i.e., physical health and mental health) through 8 components: physical performance, physical, pain, mental health, general health status, vitality, mental performance and social function. ii. 36 items iii. Information not provided, except that the instrument has a score that varies between 0 and 100 points, and higher values in the physical/mental dimension indicate better physical and mental health functioning.	i. Information not provided, but the authors refer to the original version (Ware et al., 1993). ii. Information not provided, but the study was conducted in Portugal.	i. Self-reported questionnaire ii. Information not provided	Cronbach’s alpha was 0.94, and the omega was 0.99.	Information not provided.
	Uyar et al. (2019) [87*]			Together with other instruments, the SF-36 was used to assess the impact of the Dementia Care and Support Program on caregivers and patients with dementia.	i. Quality of Life Scale SF36 comprises eight subdimensions and defines two summary areas: mental and physical health. ii. 36-items iii. The total score can be 0–100	i. Information not provided. ii. Turkish	i. Self-reported questionnaire ii. Information not provided	In this study, SF-36 had a Cronbach’s alpha ranging from 0.73 to 0.88.	Information not provided.
	Wang et al. (2020) [88*]			SF-36 examined how job demands influenced QoL for employed family caregivers of older adults with dementia in Taiwan.	i. Information not provided ii. 36-items iii. Scores range from 0 to 100.	i. QoL was operationally defined by the Taiwan version of the Medical Outcomes Study Short Form-36 Scale ii. Chinese/Taiwanese	i. Self-reported questionnaire ii. Information not provided	Cronbach’s alpha of 0.93.	Information not provided.
SIDECAR (Horton et al. 2021) [63*]				SIDECAR (Three Scales measuring the Impact of Dementia on CARers) is developed from other QoL needs-led questionnaires to measure QoL of the carers of a person with dementia. SIDECAR provides a theoretically based needs-led QoL profile specifically for dementia carers.	i. Three instruments/domains SIDECAR-D, SIDECAR-I, SIDECAR-S ii. SIDECAR-D = 18 items SIDECAR-I = 10 items SIDECAR-S = 11 items iii. 2	i. Instrument developed from comprised qualitative interviews (SIDECAR item pool T1, T2, T3) At T1, *N* = 570 (566 valid) T2, *N* = 100 (100 valid) T3, *N* = 173 (172 valid) ii. English	i. Self-reported questionnaire pack at three time points: Time 1 (T1), following consent; Time 2 (T2), 2–4 weeks later; and Time 3 (T3), for a subsample (due to time constraints), 6 months after Time 1. ii. Information not provided	Cronbach’s alpha for SIDECAR-D = 0.83 SIDECAR-I = 0.70 SIDECAR-S = 0.81 Test-retest reliability ICC ≥ 0.85.	Convergent validity (SWEMWBS): 0.4 to 0.57, indicating moderate convergent validity Convergent validity (EQ-5D VAS): 0.21 to 0.23, suggesting weak convergent validity.
WHOQOL-BREF	Hu et al. (2023) [64*]			The study adopted a secondary data analysis using data collected in a prior methodological study from Chang et al. (2016) that assessed the WHOQOL‑BREF in populations other than caregivers of people with dementia. Two general items were not included in any domain in the Taiwanese WHOQOL‑BREF. Therefore, the Taiwanese WHOQOL‑BREF contains 28 items, with two being culturally specific. One item is embedded in the social domain, assessing respect, and another item is embedded in the environmental domain, assessing eating.	i. Four domains ii. 28 items iii. Five‑point Likert scale converted into a 0–20 scale for each domain	i. Two general items were not included in any domain in the Taiwanese WHOQOL‑BREF ii. Mandarin or Taiwanese Chinese	i. Self-reported questionnaire ii. Information not provided	Cronbach’s alpha: physical; 0.80, social QoL; 0.71, psychological QoL; 0.71, social QoL; 0.50 and environmental QoL; 0.65 Total (alpha = 0.873).	Information not provided.
	Kaewwilai et al. (2022) [67*]			To assess QoL over the past 2 weeks using the brief version of the WHO Quality of Life Instrument (WHOQOL-BREF),	i. Four domains: physical health, psychological health, social relationships and environmental, with one additional item for general QOL and one item for health-related QOL. ii. 26 items iii. Five‑point Likert scale converted into a 0–20 scale for each domain. The Total QOL score is the summation of all domain scores and two global item scores. The sum scores are classified into three QOL groups: poor, moderate, and good QOL.	i. Brief version of the World Health Organization Quality of Life Instrument (WHOQOL-BREF) ii. Thai	i. Self-reported questionnaire ii. Information not provided	Cronbach’s alpha was 0.92, and each domain was above 0.59.	Information not provided.
	Kalaitzaki et al. (2022) [68*]			The WHOQoL-BREF was used to measure caregivers’ QoL	i.Four domains: Physical health, psychological health, social relationships, and environment ii. 26 items iii. Five-point scale ranging from 1 to 5 to produce a total score with a potential range of 24–120	i. Information not provided ii. Greek	(i) and (ii) Information not provided	Cronbach’s alpha was 0.881.	Information not provided
	Magteppong et al. (2021) [75*]			The World Health Organization’s Quality of Life – Thai is the Thai version of the shortened World Health Organization Quality of Life (WHOQOL-BREF).	i. Four domains: physical health, psychological health, social relationships, og environmental Health ii. 26 items in total. Each domain has questions designed to measure the QoL in that area, each containing seven items (except for the social relationships domain, which has three items). Additionally, two general questions assess overall quality of life and health, comprising 26 items. iii. Five-point Likert scale for its response categories: “Not at all”, “A little”, “A moderate amount”, “Quite a lot», and “An extreme amount”. The total score range is 26–130 points. A high score represents a high quality of life	i. Information not provided. ii. Thai	i. Self-reported questionnaire ii. Information not provided	Cronbach’s alpha was 0.96.	Information not provided.
	Meichsner et al. (2019) [74*]			The WHOQoL-BREF was modified to assess participants’ QoL over the last 2 weeks.	i. Four domains: Physical health, psychological health, social relationships, and environment ii. 26 items. Overall QoL and satisfaction with general health (Overall QoL and Health; “How would you rate your quality of life?”, “How satisfied are you with your health? iii. Information not provided. Scores were transformed into a scale ranging from 0 to 100.	i. Brief version of the World Health Organization Quality of Life Instrument ii. German	(i) and (ii) Information not provided	Cronbach’s alpha: Physical health, 0.83; psychological health, 0.81; social relationships, 0.58; environment, 0.72; overall, 0.52; overall QoL and satisfaction with general health (Overall QoL and Health; 0.52)	Information not provided.

*APLIQue* The Alzheimer’s Patient Partners Life Impact Questionnaire, *ASCOTCarer* The Adult Social Care Outcomes Toolkit for Carers, *CASP-16* The Control, Autonomy, Self-Realization, and Pleasure Scale, *C-DEMQOL* The Caregiver Dementia Quality of Life Measurement, *CQoL-ND* The Caregiver Quality of Life Index – Neurocognitive Disorder, *DQOL-OC* The Dementia Quality of Life Scale for Older Family Carers, *EQ-5D* The European Quality of Life – Five Dimensions, *EQ-5D-3L* The European Health-related Quality of Life – Five Dimensions with three response levels, *EQ-5D-5L* The European Health-related Quality of Life – Five Dimensions with five response levels, *FQOL Scale* The Beach Center Family Quality of Life Scale, *FQOLD* The Family Quality of Life in Dementia Scale, *HRQOL* The Health-Related Quality of Life Scale, *LTCQ-Carer* The Long-Term Conditions Questionnaire for Carers, *PTQL* The Pictorial Thai Quality of Life, *QOL-AD* The Quality of Life in Alzheimer’s Disease, *SCQOL-D-15* The Short Form of the Singapore Caregiver Quality of Life Scale – Dementia, *SCQOLS-D* The Caregiver-targeted Quality of Life Measure, *SIDECAR* The Scale Measuring the Impact of Dementia on Carers, *SF-12* The 12-item Short Form Health-related Quality of Life Survey, *SF-36* The 36-item Health-related Quality of Life Survey, *WHOQOL-BREF* The World Health Organization Quality of Life - Brief Version

*Development means the authors’ modification of the instruments

## Results

This scoping review included 34 studies, covering 21 different instruments used to assess QoL in informal caregivers of people with dementia.

### Characteristics of the included studies

Although all the included studies are quantitative in research design, they differ in terms of the year of publication, country, aim, sample, and the instrument used to measure QoL in informal caregivers of people with dementia.

### Year of publication

**Fig. 2:**
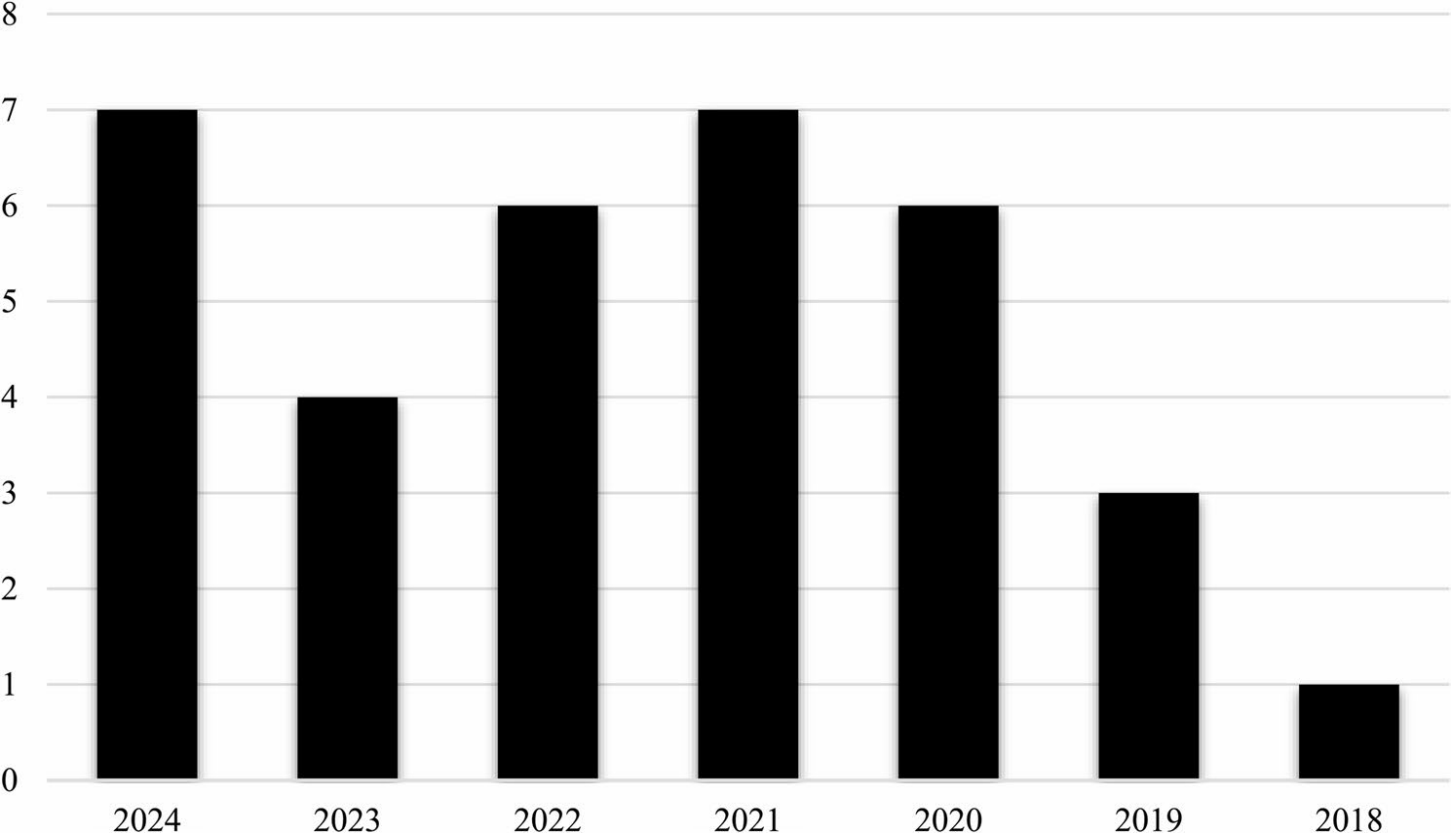
Number of publications per year

The studies in the scoping review cover six years, from 2018 to 2024, with a peak of seven studies published in 2021 and 2024, as shown in Fig. 2.

### Country of origin

**Fig. 3:**
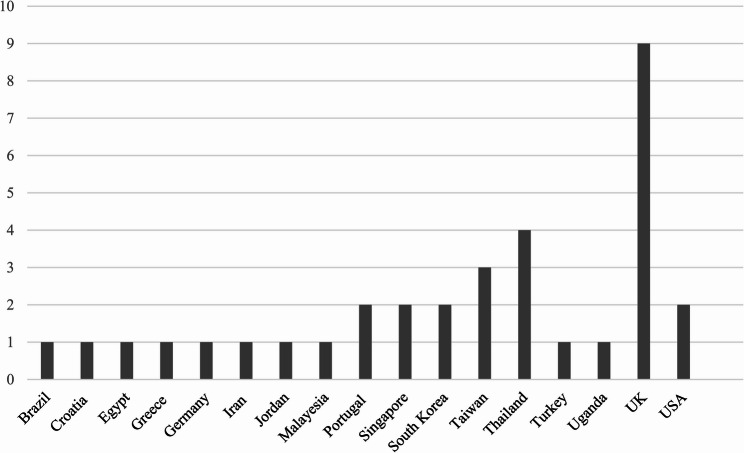
Number of articles published per country

As shown in Fig. 3, the studies were carried out across 17 countries worldwide, including nine in the UK.

### Research design

Among the 34 studies, 18 used a cross-sectional design [57*, 58*, 61*, 64*, 65*, 66*, 68*, 69*, 70*, 73*, 77*, 78*, 79*, 81*, 82*, 85*, 86*, 88*]. Table 1 provides an overview of the studies’ designs, summarising their characteristics and types.

### Study population

Based on the baseline data from the included studies, the total number of informal caregivers across all 34 studies was 6,351, with figures ranging from 61 [87*] to 566 [63*] persons. The proportion of female participants varied from 46.7% [88*] to 87.2% [67*]. The age of informal caregivers ranged from 20 years [57*] to 90 years [83*]. Most studies focused on individuals who provide care to family members living with dementia, using terms such as “caregiver”, “family carers”, “carers”, “informal caregivers”, “family carers”, or “unpaid carers”. Several studies lacked information regarding the relationship between informal caregivers and their family members with dementia, as well as whether the caregiver resided with the person with dementia. However, those who did provide this information indicated that most informal caregivers were adult children or children-in-law.

### Instruments used to measure QoL in informal caregivers of people with dementia

Across the 34 studies included in this scoping review, 21 instruments were identified. Four of these were generic (CASP-16, SF-12, SF-36, and WHOQOL-BREF), while 17 were disease- and population-specific. Three instruments were utilised in multiple studies: the WHOQOL-BREF, [ the C-DEMQOL, ] and the SF-36. One instrument, the QOL-AD, was employed in two studies [69*, 81*]. A detailed description of the instruments is provided in Table 2.

The most used instruments were the C-DEMQOL (Carer-DEMentia Quality of Life), the SF-36 (The Short Form 36 Health Survey Questionnaire), and the WHOQOL-BREF (The short form World Health Organization Quality of Life (WHOQOL-100)).

The C-DEMQOL, used in five of the included studies [41*, 60*, 61*, 62*, 84*], was designed to measure QoL in family caregivers of people with dementia [41*]. It can be administered by researchers or completed independently. The instrument includes 30 questions to assess the caregivers’ QoL, divided into five categories, each examining factors that influence QoL.

The SF-36 was used in five studies [72*, 73*, 77*, 87*, 88*]. It indicates the health status of specific populations, helps in service planning, and measures the impact of clinical and social interventions. It was designed to assess eight health concepts that comprehensively measure an individual’s health-related QoL [39]. Culture-specific data are required to calculate SF-36 norm-based scores.

The WHOQOL-BREF was also utilised in five studies [64*, 67*, 68*, 74*, 75*] . This instrument is a condensed version of the World Health Organisation Quality of Life (WHOQOL-100) instrument. It is a widely adopted instrument for evaluating an individual’s QoL across different cultures and settings. Developed by the WHOQOL Group in 1996, this instrument is applicable to various populations, including those with health conditions and the general public [16]. The WHOQOL-BREF consists of 26 items, down from the original 100-item WHOQOL.

The Quality of Life in Alzheimer’s Disease (QOL-AD) was used in two studies [69*, 81*], which includes 13 items covering various aspects of life, such as physical health, energy, mood, memory, family relations, and social activities. The instrument was initially developed to assess the QoL in individuals with Alzheimer’s disease and caregiver reports of patient QoL [92].

### Instrument domains

Table 2 presents the characteristics of the instruments used to measure QoL in informal caregivers of people with dementia. Each instrument targets different domains tailored to its specific focus, such as the needs of informal caregivers in APPLIQue [76*], aspects of life most relevant to informal caregivers in ASCOTCarer [86*], or dimensions like health, emotional well-being, social functioning, and general health perceptions in HRQOL [65*]. Despite this variation, most instruments that provide information about their domains and items focus on physical, psychological, and social aspects. The number of domains varies widely, from three in SIDECAR [63*] to 13 in QOL-AD [69*]. The number of items within these instruments also varies significantly, from as few as five in EQ-5D-3 L [82*] to 67 in HRQOL [65*].

To effectively capture the experiences and perspectives of informal caregivers, most instruments typically consist of graded Likert scales or yes/no answer options. Using a Likert scale, respondents can provide nuanced assessments of their perceived Qol. For example, the FQOLD [58*] includes statements such as “My family has the support needed to manage our feelings” and “My family gets medical care when needed,” with response options rated on a 4-point Likert scale. Similarly, the WHOQOL-BREF [74*, 75*] asks questions like “How satisfied are you with your health?” and “How would you rate your quality of life?” with response choices based on a five-point Likert scale.

### Validity and reliability of the QoL instruments reported in the included studies

One of the inclusion criteria for the studies was that they must include one or more instruments used to measure QoL in informal caregivers of people with dementia, as well as report at least one psychometric property for the instrument used to measure QoL (e.g., content validity, reliability). Only nine [41*, 57*, 60*, 63*, 71*, 76*, 83*, 85*, 86*] of the 34 included studies described results on the content validity scores of the instruments they used for informal caregivers. Some of these studies, such as those by Brown et al. [41*] and Horton et al. [63*], employed rigorous instrument development processes, which involved generating items from qualitative data and conducting individual interviews to ensure the relevance and clarity of the items. Similarly, Cheung et al. [59*] and Lee et al. [71*] demonstrated strong methodological approaches by engaging local informal caregivers in Singapore while developing the SCQOLS-D and its short form, ensuring cultural relevance and comprehensiveness. The ASCOT-Carer tool, evaluated by Silarova et al. [86*], also included qualitative testing with unpaid caregivers to ensure content validity. Two other studies, McKenna et al. [76*] and Potter et al. [83*], briefly described the involvement of informal caregivers in item selection. However, limited details on how comprehensiveness or comprehensibility was evaluated are provided. For example, neither Alzaben et al. [57*] nor Sittironnarit et al. [85*] reported formal assessments of content validity. While language translation or previous use in the country may suggest some degree of face validity, relevance, comprehensiveness, and comprehensibility specific to informal caregiving were not evaluated. Although some studies reported adequate or strong content validity based on informal caregivers, the variation in depth and transparency of reporting suggests inconsistency in how content validity was assessed across studies. This may indicate that generic instruments, such as WHOQOL-BREF, may not fully capture the specific experiences of informal caregivers of people with dementia. Their structural validity and relevance for this population should be carefully evaluated before use, or at least their structural validity should be assessed and adjustments considered for informal caregivers of people with dementia.

Reliability is assessed, according to Taber [32], by researchers using various qualitative descriptors to interpret Cronbach’s alpha (α) values, which range from excellent (0.93–0.94) to not satisfactory (0.4–0.55) and low (0.11). McDonald’s omega (ω) is also a reliability coefficient used to estimate an instrument’s internal consistency. It is regarded as a more robust alternative to Cronbach’s alpha because it does not assume equal factor loadings among items [76*]. The general interpretation of omega mirrors that of Cronbach’s alpha, ranging from excellent reliability (ω ≥ 0.90) to poor reliability (ω < 0.60). The figures reported in the included studies ranged from not satisfactory (Cronbach’s alpha 0.52), potentially indicating substantial measurement error for the WHOQOL-BREF depending on the sample examined [74*], to relatively high internal reliability (Cronbach’s alpha > 0.70) for the ASCOTCarer instrument [86*], or high internal consistency (Cronbach’s alpha of 0.96) in the Thai version of the WHOQOL-BREF [75*]. Scores on McDonald’s omega (ω) were provided in three studies [41*, 60*, 77*] for two instruments, C-DEMQOL and SF-36, and ranged from ω = 0.99 [77*] to ω = 0.97 [41*, 60*].

Regarding the method of questionnaire administration, 20 studies [59*, 63*, 64*, 65*, 66*, 67*, 69*, 71*, 72*, 75*, 77*, 78*, 80*, 82*, 83*, 85*, 86*, 87*, 88*, 89*] used a self-reported questionnaire. Seven studies [41*, 60*, 61*, 62*, 76*, 79*, 84*] , employed interviews to collect data. Seven studies [57*, 58*, 68*, 70*, 73*, 74*, 81*] did not specify the administration method. Only four studies [41*, 61*, 65*, 82*] reported the time taken to administer the questionnaire.

## Discussion

This scoping review aimed to identify and map the instruments used to measure QoL in informal caregivers of people with dementia. To our knowledge, this is the first scoping review that provides an overview of the ‘status quo’ regarding the instruments currently employed in clinical practice and research for this population since Dow et al. [20] presented results from their systematic review. Based on the results of our review, there are specific challenges in reaching consensus on which instrument best covers the intended content.

The current review identified 34 studies presenting 21 instruments to assess QoL in informal caregivers of people with dementia, including generic-, disease- and population-specific measurements. The most notable observation is the large number of instruments used to measure QoL among informal caregivers of people with dementia over eight years (2016–2025). Some instruments, such as SIDECAR [63*] and SCQOLS-D [59*] and SCQOLS-D-15 [71*] , were recently explicitly developed to measure QoL in informal caregivers of people with dementia and showed promising results regarding relevance and comprehensiveness. Further validations from several other studies would significantly improve the quality of evidence. Others, such as APPLIQue [76*] and PTQL [85*], were translated and validated in multiple languages – APPLIQue in English, German, Italian, and Spanish and PTQL in Thai – to better reflect the populations and contexts in which they were used.

This scoping review emphasises that there is no ‘one size-fits-all’ approach to selecting an instrument to measure QoL in informal caregivers of people with dementia. Instead, various instruments are available, each with different strengths and limitations concerning their utility. The choice of instrument will therefore depend on several factors, particularly the purpose of the study, the progression of dementia in the family member, and their personal standards and concerns in life. Consequently, using an instrument to measure QoL requires careful consideration of multiple factors and prerequisites.

First, the instrument should be relevant to the construct of QoL and cover what is essential for the targeted population in their living environment [16, 34, 94, 95]. The informal caregivers of people with dementia are in a special and, for many, demanding situation that influences their perceived Qol. As dementia symptoms develop, the different stages of the condition’s severity, both in terms of functional dependence and behaviour changes, may have a crucial impact on the caregiver [37]. Among the symptoms of dementia, neuropsychiatric symptoms are known to increase informal caregivers’ experience of burden more than other symptoms [96].

Secondly, there are conditions related to the informal caregivers themselves that guide the content of the instruments. Therefore, to make a clinical judgement of QoL, the instrument must include information about caregiver and kinship differences [97]. Spouses of older persons with dementia, adult children, young people, or children of parents with early-onset dementia may experience the burden differently [98]. Moreover, since the samples in each study included in the scoping review predominantly consist of women, they may experience caregiver burden differently from how men perceive it. Results from a systematic review [99] demonstrate a higher reported burden or care-related distress among female caregivers. Therefore, guidance regarding the content of the QoL instruments should be validated according to the target population group.

Recent literature has shown that informal caregivers experience burden, stress, and burnout as a result of the progression of the dementia in the person they are caring for [100, 101]. This affects how they perceive their QoL [11]. Other studies have also found that informal caregivers of people with dementia are more likely to suffer from various physical and mental conditions [11, 102] and feelings of blame, guilt, loneliness, and social isolation [103–105].

The loss experienced by informal caregivers is described as a grieving process as the dementia worsens and affects the person’s cognitive, behavioural, and functional abilities [106]. In cases of dementia, informal caregivers face a unique type of loss, as the person with dementia remains physically present but is no longer able to engage meaningfully on a psychological level [107 ]. All these factors lead to informal caregivers experiencing psychological distress, physical health issues, social isolation, and financial difficulties [108 ]. Therefore, it is essential to understand how these aspects of informal caregivers align with the content of QoL instruments.

Instruments used in research and clinical practice depend on the methodological rigour of QoL instruments. When selecting a QoL instrument to measure the QoL in informal caregivers of people with dementia, several measurement properties must be considered to assess how well an instrument encompasses all relevant aspects of the construct to be measured [28]. However, the relevance and importance of these measurement properties are thoroughly discussed in several studies [38,59*, 71*]. Here, QoL is a theoretical concept that, in particular, cannot usually be measured directly [24]. Unfortunately, there is no comprehensive theory of QoL; however, the definitions provided by the WHOQOL Group [16] and health-related QoL models are sometimes used [109].

The WHOQOL Group’s [16] definition emphasises that people’s relationships with their goals, expectations, standards, and concerns in life, as well as with their living arrangements, are important when considering their Qol. Therefore, according to Terwee et al. [93], the instrument should be relevant to the construct of QoL in the targeted population. Additionally, the instrument should be comprehensive for informal caregivers and be understood as intended [34, 94, 95]. Consequently, a clinical judgement of the caregiver is necessary when using an instrument to assess their QoL while they provide care to a family member with dementia.

The WHOQOL Group’s [16] description of QoL as ‘individuals’ perception of their position in life’ indicates that all aspects of life are included in the person’s assessment of their Qol. This broad description encompasses health profiles and generates health utilities [36], which is quite useful. Most instruments identified in the current scoping review are either generic or caregiver related. Nonetheless, the studies included also provide examples of instruments measuring both general and overall QoL [69*, 75*, 74*, 60*].

Conversely, disease- and population-specific instruments focus on issues related to a single disease condition, patient group, or aspects of physical or mental functioning [27]. Informal caregivers might not be in poor health, but taking on the caregiver role can lead to significant emotional, physical, and psychological challenges [11, 102 , 105 ]. Consequently, they often experience co-morbidities or even multi-morbidities. Therefore, using a disease-specific instrument in research studies is not recommended [37]. However, in clinical practice, disease-specific instruments could be useful for assessing the impact of the disease on QoL in informal caregivers. Nonetheless, neither disease-specific nor generic instruments are sufficiently sensitive to identify the psychological effects or even the positive aspects of caregiving [37].

Based on our review of the instruments’ subscales or domains, the results revealed diverse content and a lack of consensus on what the instruments should measure. The most common domains appeared to be physical health, psychological health, social relationships, and environment. These domains are represented in the WHO’s Quality of Life short version (WHOQOL-BREF), which was used in five studies [64*, 67*, 68*, 74*, 75*] . In addition to these domains, this instrument includes two items that assess overall QoL and general health. As shown in this review, the WHOQOL-BREF has been translated into several languages across the included studies; however, only minimal measurement properties, typically limited to Cronbach’s alpha, were reported. Other instruments to measure QoL include SF-36, a generic instrument appearing in different versions with various domain counts in five studies [72*, 73*, 77*, 87*, 88*] , and C-DEMQOL [41*, 60*, 61*, 62*, 84*] , a disease- and population-specific instrument developed to assess QoL in informal caregivers of people with dementia. Additional domains, such as social relationships, financial resources, and suitable housing, can be essential to the caregiver situation [109]. However, the included studies varied in their reporting of psychometric properties, as well as in the information provided on ease of administration, scale dimensions, and other relevant characteristics. Although some studies [41*, 57*, 58*, 60*, 66*, 75*, 76*, 77*, 78*, 80*, 82*, 83*, 84*, 88*] reported the highest levels of internal consistency, with Cronbach’s alpha values exceeding 0.90 for the instruments used, further evaluation of other measurement properties was also recommended. This indicates a need for a more comprehensive assessment of the instruments. Additional validation is necessary to evaluate instruments designed for the specific group of informal caregivers before they can be endorsed. Another issue is whether there is a need for additional instruments to assess QoL or simply to refine the existing ones [59*].

Dow et al. [20] recommended the Carer Well-being and Support questionnaire (CWS) for healthcare and social care professionals because this instrument includes a needs assessment component. However, Dow et al. [20] mentioned that all instruments included in their review would benefit from more rigorous evaluation of their measurement properties, a recommendation that also applies to our study.

The challenges faced by informal caregivers, as described in the studies included in the current scoping review, raise questions about defining the optimal content of the QoL measure. Content validity refers to the extent to which an instrument accurately reflects the construct it aims to measure [94], which, in this case, is the QoL among informal caregivers of people with dementia. Throughout the reviewed studies, there is notable variation in the focus on establishing or reporting the content validity of the instruments, a factor that is especially crucial when developing a new instrument.

Two studies [41*,^63^*] specifically developed new QoL tools tailored for informal caregivers of people with dementia (C-DEMQOL and SIDECAR, respectively). These studies followed thorough development processes, including extensive stakeholder engagement (with caregivers, clinicians, and experts), individual interviews, and pilot testing. As a result, the content validity of these tools is likely to be strong, with items reflecting areas particularly relevant to the caregiving experience, such as role strain, emotional well-being, social support, and the influence of dementia-specific behaviours. Similarly, two other studies demonstrated good practices when developing and validating the SCQOLS-D [59*] and the SCQOLS-D-15 [71*] by adapting their instruments through qualitative input from informal caregivers. This culturally rooted approach enhances content validity by ensuring that items are pertinent to caregivers within that specific socio-cultural setting.

On the other hand, several studies [64*, 67*, 68*, 72*, 73*, 74*, 75*, 77*, 87*, 88*, 89*] used generic QoL instruments (e.g., SF-12, SF-36, WHOQOL-BREF) or caregiver burden measures as proxies for QoL, such as in Alzaben et al. [57*], using EQ-5D-5 L, and in Gumikiriza-Onoria et al. [61*], using C-DEMQOL. While these instruments can be useful for broader health or psychological assessments, they often lack items specific to the unique challenges of dementia caregiving. This raises concerns about content validity, as these instruments may not fully capture the multidimensional burden and psychosocial effects experienced by caregivers.

Furthermore, although QoL was evaluated in several studies [61*, 66*, 68*, 78*], the tools used were either not specified or not tailored for the target population. This lack of clarity reduces confidence in the validity of the results, especially in cross-cultural or minority group contexts where caregiving experiences can vary greatly.

The study by Silarova et al. [86*] highlights the assessment of the ASCOT-Carer instrument for informal caregivers of individuals with dementia in England, where content validity, including the relevance of domains and clarity of language, was formally evaluated. Similarly, Potter et al. [83*] used the LTCQ-Carer instrument, which showed a strong dedication to ensuring that the measure reflected the real experiences of informal caregivers through thorough validation procedures.

Overall, while some studies demonstrated high standards in developing dementia-specific and culturally responsive QoL instruments, thereby improving content validity, many others used generic instruments without proper adaptation or failed to document the process of ensuring content relevance.

Reliability is a key psychometric property that pertains to the consistency and stability of a measurement instrument [29]. In the context of QoL instruments for informal caregivers of people with dementia, several types of reliability are particularly important – internal consistency, test-retest reliability, and inter-rater reliability – each representing a different facet of measurement accuracy. During the full-text review, some studies did not report at least the internal consistency of the tools used. The absence of reported internal consistency may suggest limitations in methodological rigour, as it hampers the evaluation of the instruments’ reliability and reduces confidence in the stability and coherence of the reported findings. As a result, those studies were excluded.

Across the studies included in this scoping review, internal consistency, often assessed with Cronbach’s alpha, was the most frequently reported form of reliability. Instruments such as C-DEMQOL [41*, 60*, 61*, 62*, 84*], SIDECAR [63*], and the SCQOLS-D and SCQOLS-D-15 [59*, 71*] demonstrated strong internal consistency across multiple domains, indicating that their items consistently measured cohesive constructs related to caregiver QoL.

Test-retest reliability, which evaluates the consistency of responses over time, was less frequently reported [29]. However, its absence is notable, especially for instruments intended for longitudinal assessment or intervention studies [60*, 63*, 72*, 74*, 81*]. A lack of test-retest data limits our understanding of whether these instruments can reliably detect changes over time in caregivers’ QoL, which is crucial for assessing the effectiveness of interventions.

Inter-rater reliability was not applicable or reported in most studies, as the QoL instruments were generally self-reported by caregivers. However, this aspect may require more focus in future research for instruments involving interviewer-administered formats or proxy responses, such as in two COVID-19 pandemic-related studies [60*, 62*, 84*].

Some studies [74*, 77*, 89*] used well-established generic instruments (e.g., SF-12, SF-36, WHOQOL-BREF), which have strong general psychometric credentials but were not always validated within the specific cultural or informal dementia caregiving context. This raises questions about whether reported reliability estimates are applicable to diverse caregiving populations. Conversely, culturally adapted instruments, such as those used in studies from Thailand [75*], South Korea [70*], and Taiwan [72*], sometimes reported high internal consistency; however, other aspects of reliability were not always addressed.

Based on the findings of the current study, we recommend further assessment of the included instruments, as well as those developed in the future. When measuring QoL in informal caregivers of people with dementia, we advise using an instrument that considers various factors, including caregivers’ age, physical and mental health, economic situation, the stage of dementia in the person they care for, the presence of neuropsychiatric symptoms, and other relevant conditions. The selection of an instrument will depend on several aspects, such as the purpose of data collection and use, available resources, and the specific local context. Environment or context plays a crucial role in shaping the theoretical framework and ensuring content validity. For example, McKenna et al. [76*] designed the APPLIQue instrument specifically for use in the UK, making it the most suitable choice for assessing content validity among informal caregivers of people with Alzheimer’s Disease in the UK. If APPLIQue is to be utilised for evaluating quality of life in informal caregivers of people with dementia in other countries, its cross-cultural validity should be established beforehand.

Finally, when selecting an instrument, it is important to ensure it meets fundamental measurement properties. For developing a new instrument, content validity is vital, while for existing instruments, internal consistency is crucial, as indicated by Cronbach’s alpha and/or McDonald’s omega. Based on this scoping review’s findings, among the instruments listed, SIDECAR [63*] and SCQOLS-D [59*] seem promising due to their specificity for informal caregivers of people with dementia. The APPLIQue [76*] is especially suitable for assessments within the UK. Additionally, C-DEMQOL [41*, 60*, 61*, 62*, 84*] stands out as a disease- and population-specific instrument, and WHOQOL-BREF [64*, 67*, 68*, 74*, 75*] provides a widely used, generic option that covers multiple QoL domains.

### Strengths and limitations

Similar to other scoping reviews, the results from this review depend on the quality of the existing literature and how detailed and comprehensive the information is. One limitation may be the exclusion of studies that did not report at least one psychometric property for the instrument used to measure Qol. Additionally, we have not assessed whether the instruments were validated for a specific gender in the target population or whether the instruments are freely accessible or require payment, which may be seen as a limitation in our presentation.

Although the database search was recently updated, there remains a risk that relevant studies presenting other instruments have not been captured. This review focused solely on articles published in English or Scandinavian languages, which may have led to the exclusion of studies in other languages and potentially caused an underestimation of the available evidence. Nevertheless, the selection of databases, the search strategy, and the application of inclusion criteria were carefully designed and guided by prior research and established literature to ensure the broadest possible range and diversity of relevant studies, thereby fulfilling the aim of the scoping review.

One notable strength is that the scoping review adhered to the five steps outlined in the JBI Manual [49], providing a solid methodological foundation for the review. Another strength is that we included studies that used and presented both generic and/or disease- and population-specific instruments, thereby demonstrating the diverse range and variety of tools employed to measure QoL in informal caregivers of people with dementia, reflecting the multifaceted nature of caregiving experiences and measurement approaches. Consequently, it is difficult to draw definitive conclusions about which instrument is most suitable for assessing QoL in informal caregivers of people with dementia in clinical settings and future research.

### Clinical implications

The results of this scoping review have significant clinical implications for assessing QoL in informal caregivers of people with dementia. Although numerous instruments are available to measure QoL in this group, the absence of tools specifically developed or validated for dementia caregiving contexts restricts their clinical usefulness.

The significant variability in content, structure, and psychometric quality across instruments emphasises the risk of choosing tools that may not fully capture the lived experiences or key challenges faced by informal caregivers. Content validity of PROMs can be assessed by consulting informal caregivers, patients, and/or professional experts regarding the relevance, comprehensiveness, and clarity of the items, response options, and instructions. Such an evaluation is essential for ensuring that the instruments are appropriate for their specific caregiving contexts.

Based on the findings of this scoping review, QoL instruments for informal caregivers of people with dementia should ideally assess domains such as emotional well-being, social support, caregiver burden, role satisfaction, and the impact of caregiving on personal life and identity. These domains are frequently addressed in instruments that demonstrate stronger content validity [59*, 83*, 86*] . Ensuring that these constructs are covered can enhance the clinical utility and relevance of QoL assessments in this population. Clinicians and researchers should exercise caution when selecting QoL instruments and ensure that the chosen instruments align closely with the specific caregiving context and assessment objectives. Importantly, given the absence of a superior instrument, clinicians and researchers may consider supplementing existing instruments with additional assessments or interviews that address overlooked but clinically relevant domains. Ultimately, there is a pressing need to develop and validate informal caregiver-specific QoL instruments to support more accurate needs assessments, care planning, and outcome monitoring in dementia care.

## Conclusion

Several instruments are available to assess QoL in informal caregivers of people with dementia, but few have been specifically designed for this group. Overall, there is limited evidence regarding the content validity and internal consistency of these tools. The findings of this scoping review suggest that none of these instruments can be recommended as superior for use among informal caregivers of people with dementia.

The scoping review revealed considerable variability in the scales, domains, items, response categories, administration times, and modes of the instruments. Instruments specifically designed for or validated in informal caregiver populations, such as the APPLIQue , ASCOT-Carer, LTCQ-Carer, and SCQOLS-D, showed stronger support for content validity because their items more directly reflect caregiving-related challenges and experiences. Conversely, generic instruments like the WHOQOL-BREF included several items that might be less relevant or meaningful in the context of informal dementia caregiving (e.g., satisfaction with appearance). These frequently lacked documented formal assessments of content validity within this population. These findings indicate that the use of generic instruments should be approached cautiously, and future research should focus on selecting instruments with confirmed relevance to the caregiving context. Therefore, future studies should carefully assess the content of these tools in relation to the study’s purpose, whether for research or clinical assessments. Qualitative exploration, caregiver participation, and cross-cultural validation will be crucial for accurately and ethically measuring QoL in this population. Additionally, for QoL instruments to be truly reliable and applicable across research and clinical settings, future research should aim to conduct comprehensive testing across diverse caregiver groups and environments.

Instruments chosen to assess the QoL of informal caregivers of people with dementia should specifically target the life areas most impacted in this group. Based on current evidence, no single instrument can be confidently recommended. We strongly call for more thorough evaluations of the psychometric properties of these instruments, with content validity and internal consistency as the minimum standards for reporting. These findings highlight the urgent need for psychometrically sound instruments designed specifically to measure QoL in informal caregivers of people with dementia.

## Supplementary Information

Supplementary Material 1.

Supplementary Material 2.

Supplementary Material 3.

## Data Availability

All data generated or analysed during this study are included in this published article and its Supplementary file.

## Abbreviations

APLIQue: The Alzheimer’s Patient Partners Life Impact Questionnaire
ASCOTCarer: The Adult Social Care Outcomes Toolkit for Carers
CASP-16: The Control, Autonomy, Self-Realization, and Pleasure Scale
C-DEMQOL: The Caregiver Dementia Quality of Life Measurement
CQoL-ND: The Caregiver Quality of Life Index – Neurocognitive Disorder
DQOL-OC: The Dementia Quality of Life Scale for Older Family Carers
EQ-5D: The European Quality of Life – Five Dimensions
EQ-5D-3L: The European Health-related Quality of Life – Five Dimensions with three response levels
EQ-5D-5L: The European Health-related Quality of Life – Five Dimensions with five response levels
FQOL Scale: The Beach Center Family Quality of Life Scale
FQOLD: The Family Quality of Life in Dementia Scale
HRQOL: The Health-Related Quality of Life Scale
LTCQ-Carer: The Long-Term Conditions Questionnaire for Carers
PTQL: The Pictorial Thai Quality of Life
QOL-AD: The Quality of Life in Alzheimer’s Disease
SCQOL-D-15: The Short Form of the Singapore Caregiver Quality of Life Scale - Dementia
SCQOLS-D: The Caregiver-targeted Quality of Life Measure
SIDECAR: The Scale Measuring the Impact of Dementia on Carers
SF-12: The 12-item Short Form Health-related Quality of Life Survey
SF-36: The 36-item Health-related Quality of Life Survey
WHOQOL-BREF: The World Health Organization Quality of Life - Brief Version
