# Supplementary material for: Assessing quality of life—a scoping review of studies presenting quality of life instruments for informal caregivers of persons with dementia
Source: BMC Geriatr. 2025 Nov 22;25:976. doi: 10.1186/s12877-025-06455-x (PMC12661884; doi:10.1186/s12877-025-06455-x)
Supplement: Supplementary file 1 — Supplementary Material 1. [file 12877_2025_6455_MOESM1_ESM.docx]

# Supplementary file 2 – Updated database search (as of January 2025)

### The study aims to identify and map the literature presenting quality-of-life instruments for informal caregivers of persons with dementia

### The search is updated for the period 2024-2025

### Results

The references are provided in a compressed EndNote library as an .enlx file.

| ***Databases*** | ***Results*** |
| --- | --- |
| *Medline via Ovid* | *126* |
| *Embase via Ovid* | *208* |
| *PsycINFO via Ovid* | *26* |
| *Cinahl via Ebscohost* | *86* |
| *SocIndex via Ebscohost* | *10* |
| *Web of Science (Clarivate)* | *122* |
| *Epistemonikos* | *10* |
| *Total med dubletter* | *588* |
| *Total etter automatisk dublettfjerning* | *497* |

### Search history

**Database:** Medline 1946 to January 06, 2025 (Ovid)

**Date:** 09.01.2025

**Results:** 126

| **#** | **Searches** | **Results** |
| --- | --- | --- |
| 1 | exp Dementia/ | 223782 |
| 2 | (dement* or senil* or presenil* or alzheimer*).tw,kw,kf. | 326552 |
| 3 | or/1-2 | 367658 |
| 4 | Caregivers/ | 55939 |
| 5 | (caregiver* or care giver* or caretaker* or care taker* or carer*).tw,kw,kf. | 132121 |
| 6 | ((child* or son or sons or daughter* or sibling* or brother* or sister* or wife* or wives or husband* or partner* or spous* or married* or famil* or parent* or father* or mother* or next of kin* or kinship* or significant other* or relative* or informal or unpaid) adj3 (care or caring or caregiving or care giving)).tw,kw,kf. | 105164 |
| 7 | Adult Children/ or Siblings/ or Spouses/ or Family/ or Nuclear family/ or Family relations/ or parents/ or fathers/ or mothers/ or marriage/ | 288092 |
| 8 | (caring or caregiving or care giving).tw,kw,kf. | 73122 |
| 9 | 7 and 8 | 10910 |
| 10 | 4 or 5 or 6 or 9 | 229451 |
| 11 | 3 and 10 | 21254 |
| 12 | "Surveys and Questionnaires"/ | 607973 |
| 13 | Self Report/ | 47213 |
| 14 | "Weights and Measures"/ | 2673 |
| 15 | ((caregiver* or care giver* or carer*) adj1 (Index* or instrument* or scale* or assessment* or questionnair* or measur* or survey* or test or tests or scheme* or tool* or scoring* or score*)).tw,kw,kf. | 3178 |
| 16 | ((caregiver* or care giver* or carer*) adj4 (Index* or instrument* or scale* or assessment* or questionnair* or measur* or survey* or test or tests or scheme* or tool* or scoring* or score*)).ti,kw,kf. | 2016 |
| 17 | (dement* adj2 (Index* or instrument* or scale* or assessment* or questionnair* or measur* or survey* or test or tests or scheme* or tool* or scoring* or score*)).ti,kw,kf. | 2218 |
| 18 | (sf 6 or short form 6 or sf 8 or short form 8 or sf 12 or Short Form 12 or sf 20 or short form 20 or sf 36 or short form 36 or Medical outcomes study short-form health survey).tw,kw,kf. | 41535 |
| 19 | or/12-18 | 677879 |
| 20 | "Quality of Life"/ | 298877 |
| 21 | personal satisfaction/ | 26215 |
| 22 | (quality of life or wellbeing or well being or life satis*).tw,kw,kf. | 586488 |
| 23 | or/20-22 | 662775 |
| 24 | 19 and 23 | 126350 |
| 25 | ((Quality of Life or QOL or HRQOL or EUROQOL or wellbeing or well being or (satisf* adj2 life)) adj4 (Index* or instrument* or scale* or assess* or questionnair* or measur* or survey* or test* or scheme* or tool* or scoring* or score*)).tw,kw,kf. | 147985 |
| 26 | (WHOLQOL* or WHOQOL* or WHO QOL* or DEMQOL or EQ5D* or EQ 5D* or European Quality of Life 5 Dimensions or CarerQol* or casp 19 or scales measuring the impact of dementia on carers or sidecar or QOL-AD or CQLI or QOLLTI F or (dement* specific adj (Quality of Life or QOL or HRQOL))).tw,kw,kf. | 21202 |
| 27 | or/24-26 | 223764 |
| 28 | 11 and 27 | 1685 |
| 29 | limit 28 to (danish or english or norwegian or swedish) | 1605 |
| 30 | limit 29 to yr="2024 - 2025" | 126 |

**Database:** Embase 1974 to 2025 January 07 (Ovid)

**Date:** 09.01.2025

**Results:** 208

| **#** | **Searches** | **Results** |
| --- | --- | --- |
| 1 | exp dementia/ | 485440 |
| 2 | (dement* or senil* or presenil* or alzheimer*).tw,kw. | 442268 |
| 3 | or/1-2 | 580683 |
| 4 | Caregiver/ | 131415 |
| 5 | Caregiver burden/ or Caregiver burnout/ or Caregiver support/ | 16684 |
| 6 | (caregiver* or care giver* or caretaker* or care taker* or carer*).tw,kw. | 184461 |
| 7 | ((child* or son or sons or daughter* or sibling* or brother* or sister* or wife* or wives or husband* or partner* or spous* or married* or famil* or parent* or father* or mother* or next of kin* or kinship* or significant other* or relative* or informal or unpaid) adj3 (care or caring or caregiving or care giving)).tw,kw. | 130949 |
| 8 | or/4-7 | 316702 |
| 9 | Adult child/ or Sibling/ or First-degree relative/ or Nuclear family/ or Brother/ or Sister/ or Son/ or Daughter/ or Parent/ or mother/ or father/ or Family/ or Family relation/ or Child parent relation/ or Spouse/ or Domestic partner/ or Husband/ or Wife/ or marriage/ | 540139 |
| 10 | (caring or caregiving or care giving).tw,kw. | 91130 |
| 11 | 9 and 10 | 15365 |
| 12 | 8 or 11 | 320037 |
| 13 | 3 and 12 | 32091 |
| 14 | health survey/ | 244187 |
| 15 | exp questionnaire/ | 1035531 |
| 16 | self report/ | 168897 |
| 17 | measurement/ | 105877 |
| 18 | rating scale/ | 124910 |
| 19 | scoring system/ | 340148 |
| 20 | ((caregiver* or care giver* or carer*) adj1 (Index* or instrument* or scale* or assessment* or questionnair* or measur* or survey* or test or tests or scheme* or tool* or scoring* or score*)).tw,kw. | 4713 |
| 21 | ((caregiver* or care giver* or carer*) adj4 (Index* or instrument* or scale* or assessment* or questionnair* or measur* or survey* or test or tests or scheme* or tool* or scoring* or score*)).ti,kw. | 2593 |
| 22 | (dement* adj2 (Index* or instrument* or scale* or assessment* or questionnair* or measur* or survey* or test or tests or scheme* or tool* or scoring* or score*)).ti,kw. | 2712 |
| 23 | (sf 6 or short form 6 or sf 8 or short form 8 or sf 12 or Short Form 12 or sf 20 or short form 20 or sf 36 or short form 36 or Medical outcomes study short-form health survey).tw,kw. | 64778 |
| 24 | short form 36/ or short form 12/ or short form 20/ or short form 8/ | 55261 |
| 25 | or/14-24 | 1857712 |
| 26 | "quality of life"/ | 706580 |
| 27 | satisfaction/ or life satisfaction/ | 97753 |
| 28 | (quality of life or wellbeing or well being or life satis*).tw,kw. | 850682 |
| 29 | or/26-28 | 1115276 |
| 30 | 25 and 29 | 295599 |
| 31 | "european quality of life 5 dimensions questionnaire"/ or "european quality of life 5 dimensions 3 level questionnaire"/ or "european quality of life 5 dimensions 5 level questionnaire"/ or "european quality of life 5 dimensions visual analogue scale"/ | 20056 |
| 32 | "quality of life assessment"/ | 17147 |
| 33 | "quality of life index"/ | 3369 |
| 34 | satisfaction with life scale/ | 1785 |
| 35 | ((Quality of Life or QOL or HRQOL or EUROQOL or wellbeing or well being or (satisf* adj2 life)) adj4 (Index* or instrument* or scale* or assess* or questionnair* or measur* or survey* or test* or scheme* or tool* or scoring* or score*)).tw,kw. | 231807 |
| 36 | (WHOLQOL* or WHOQOL* or WHO QOL* or DEMQOL or EQ5D* or EQ 5D* or European Quality of Life 5 Dimensions or CarerQol* or casp 19 or scales measuring the impact of dementia on carers or sidecar or QOL-AD or CQLI or QOLLTI F or (dement* specific adj (Quality of Life or QOL or HRQOL))).tw,kw. | 37929 |
| 37 | or/30-36 | 425323 |
| 38 | 13 and 37 | 3202 |
| 39 | limit 38 to (conference abstract or conference paper or "conference review") | 881 |
| 40 | 38 not 39 | 2321 |
| 41 | limit 40 to (danish or english or norwegian or swedish) | 2219 |
| 42 | limit 41 to yr="2024 -Current" | 208 |

**Database:** APA PsycInfo 1806 to December 2024 Week 5 (Ovid)

**Date:** 09.01.2025

**Results:** 26

| **#** | **Searches** | **Results** |
| --- | --- | --- |
| 1 | exp Dementia/ | 99671 |
| 2 | Alzheimer's Disease/ | 60377 |
| 3 | (dement* or senil* or presenil* or alzheimer*).tw. | 132058 |
| 4 | or/1-3 | 133134 |
| 5 | Caregivers/ | 41146 |
| 6 | Caregiver burden/ | 7919 |
| 7 | (caregiver* or care giver* or caretaker* or care taker* or carer*).tw. | 85895 |
| 8 | ((child* or son or sons or daughter* or sibling* or brother* or sister* or wife* or wives or husband* or partner* or spous* or married* or famil* or parent* or father* or mother* or next of kin* or kinship* or significant other* or relative* or informal or unpaid) adj3 (care or caring or caregiving or care giving)).tw. | 63364 |
| 9 | or/5-8 | 137596 |
| 10 | Adult offspring/ | 4801 |
| 11 | Offspring/ | 7018 |
| 12 | Sons/ | 1817 |
| 13 | Daughters/ | 3869 |
| 14 | Siblings/ | 8499 |
| 15 | Brothers/ | 630 |
| 16 | Sisters/ | 721 |
| 17 | Family/ | 66510 |
| 18 | Family relations/ | 41031 |
| 19 | Nuclear family/ | 524 |
| 20 | Parents/ | 54422 |
| 21 | mothers/ | 45829 |
| 22 | fathers/ | 12762 |
| 23 | Parent child relations/ | 34431 |
| 24 | Spouses/ | 13015 |
| 25 | Husbands/ | 2585 |
| 26 | Wives/ | 3949 |
| 27 | Significant others/ | 1597 |
| 28 | Partners/ | 7725 |
| 29 | marriage/ | 10550 |
| 30 | or/10-29 | 264081 |
| 31 | (caring or caregiving or care giving).tw. | 49957 |
| 32 | 30 and 31 | 10976 |
| 33 | 9 or 32 | 139978 |
| 34 | 4 and 33 | 15879 |
| 35 | surveys/ | 14597 |
| 36 | exp questionnaires/ | 28301 |
| 37 | self-report/ | 24389 |
| 38 | measurement/ | 64892 |
| 39 | rating scales/ | 26382 |
| 40 | Test Forms/ | 4605 |
| 41 | ((caregiver* or care giver* or carer*) adj1 (Index* or instrument* or scale* or assessment* or questionnair* or measur* or survey* or test or tests or scheme* or tool* or scoring* or score*)).tw. | 1634 |
| 42 | ((caregiver* or care giver* or carer*) adj4 (Index* or instrument* or scale* or assessment* or questionnair* or measur* or survey* or test or tests or scheme* or tool* or scoring* or score*)).ti,id. | 1103 |
| 43 | (dement* adj2 (Index* or instrument* or scale* or assessment* or questionnair* or measur* or survey* or test or tests or scheme* or tool* or scoring* or score*)).ti,id. | 1325 |
| 44 | (sf 6 or short form 6 or sf 8 or short form 8 or sf 12 or Short Form 12 or sf 20 or short form 20 or sf 36 or short form 36 or Medical outcomes study short-form health survey).tw. | 8156 |
| 45 | or/35-44 | 162250 |
| 46 | "Quality of Life"/ | 51609 |
| 47 | Health Related Quality of Life/ | 8367 |
| 48 | life satisfaction/ | 14019 |
| 49 | well being/ | 68651 |
| 50 | satisfaction/ | 14809 |
| 51 | (quality of life or wellbeing or well being or life satis*).tw. | 239611 |
| 52 | or/46-51 | 257580 |
| 53 | 45 and 52 | 18618 |
| 54 | Quality of Life Measures/ | 978 |
| 55 | ((Quality of Life or QOL or HRQOL or EUROQOL or wellbeing or well being or (satisf* adj2 life)) adj4 (Index* or instrument* or scale* or assess* or questionnair* or measur* or survey* or test* or scheme* or tool* or scoring* or score*)).tw. | 48622 |
| 56 | (WHOLQOL* or WHOQOL* or WHO QOL* or DEMQOL or EQ5D* or EQ 5D* or European Quality of Life 5 Dimensions or CarerQol* or casp 19 or scales measuring the impact of dementia on carers or sidecar or QOL-AD or CQLI or QOLLTI F or (dement* specific adj (Quality of Life or QOL or HRQOL))).tw. | 5392 |
| 57 | or/53-56 | 59373 |
| 58 | 34 and 57 | 898 |
| 59 | limit 58 to (danish or english or norwegian or swedish) | 842 |
| 60 | limit 59 to yr="2024 -Current" | 26 |

**Database:** Cinahl (EbscoHost)

**Date:** 09.01.2025

**Results:** 86

| **#** | **Query** | **Results** |
| --- | --- | --- |
| S1 | (MH "Dementia+") | 86,613 |
| S2 | TI ( (dement* or senil* or presenil* or alzheimer*) ) OR AB ( (dement* or senil* or presenil* or alzheimer*) ) | 98,170 |
| S3 | S1 OR S2 | 118,030 |
| S4 | (MH "Caregivers") OR (MH "Caregiver Burden") OR (MH "Caregiver Attitudes") OR (MH "Caregiver Support") | 60,481 |
| S5 | TI ( (caregiver* or "care giver*" or caretaker* or "care taker*" or carer*) ) OR AB ( (caregiver* or "care giver*" or caretaker* or "care taker*" or carer*) ) | 87,757 |
| S6 | TI ( ((child* or son or sons or daughter* or sibling* or brother* or sister* or wife* or wives or husband* or partner* or spous* or married* or famil* or parent* or father* or mother* or "Next of kin*" or kinship* or "significant other*" or relative* or informal or unpaid) N2 (care or caring or caregiving or "care giving")) ) OR AB ( ((child* or son or sons or daughter* or sibling* or brother* or sister* or wife* or wives or husband* or partner* or spous* or married* or famil* or parent* or father* or mother* or "Next of kin*" or kinship* or "significant other*" or relative* or informal or unpaid) N2 (care or caring or caregiving or "care giving")) ) | 66,689 |
| S7 | S4 OR S5 OR S6 | 156,836 |
| S8 | (MH "Adult Children") OR (MH "Family") OR (MH "Family Relations") OR (MH "Nuclear Family") OR (MH "Daughters") OR (MH "Siblings") OR (MH "Sons") OR (MH "Spouses") OR (MH "Parents") | 138,082 |
| S9 | (MH "Marriage") | 9,805 |
| S10 | (MH "Patient-Family Relations") | 3,133 |
| S11 | (MH "Fathers") OR (MH "Mothers") | 44,351 |
| S12 | (MH "Significant Other") | 1,294 |
| S13 | S8 OR S9 OR S10 OR S11 OR S12 | 185,605 |
| S14 | TI ( (caring or caregiving or "care giving") ) OR AB ( (caring or caregiving or "care giving") ) | 60,675 |
| S15 | S13 AND S14 | 10,023 |
| S16 | S7 OR S15 | 158,562 |
| S17 | (MH "Surveys") OR (MH "Self Report") | 247,957 |
| S18 | (MH "Questionnaires") OR (MH "Open-Ended Questionnaires") OR (MH "Structured Questionnaires") OR (MH "Scales") | 875,148 |
| S19 | (MH "Weights and Measures") | 5,360 |
| S20 | (MH "Instrument Validation") | 50,412 |
| S21 | (MH "Research Instruments") OR (MH "Caregiver Strain Index") OR (MH "Family Coping Coherence Index") OR (MH "Jalowiec Coping Scale") OR (MH "Short Form-36 Health Survey (SF-36)") | 44,486 |
| S22 | TI ( ((caregiver* or "care giver*" or carer*) N3 (Index* or instrument* or scale* or assessment* or questionnair* or measur* or survey* or test or tests or scheme* or tool* or scoring* or score*)) ) OR AB ( ((caregiver* or "care giver*" or carer*) N0 (Index* or instrument* or scale* or assessment* or questionnair* or measur* or survey* or test or tests or scheme* or tool* or scoring* or score*)) ) | 2,810 |
| S23 | TI ( (dement* N1 (Index* or instrument* or scale* or assessment* or questionnair* or measur* or survey* or test or tests or scheme* or tool* or scoring* or score*)) ) OR AB ( (dement* N1 (Index* or instrument* or scale* or assessment* or questionnair* or measur* or survey* or test or tests or scheme* or tool* or scoring* or score*)) ) | 4,001 |
| S24 | TI ( ("sf 6" or "short form 6" or "sf 8"or "short form 8" or "sf 12" or "Short Form 12" or "sf 20" or "short form 20" or "sf 36" or "short form 36" or "Medical outcomes study short-form health survey") ) OR AB ( ("sf 6" or "short form 6" or "sf 8"or "short form 8" or "sf 12" or "Short Form 12" or "sf 20" or "short form 20" or "sf 36" or "short form 36" or "Medical outcomes study short-form health survey") ) | 3,662 |
| S25 | S17 OR S18 OR S19 OR S20 OR S21 OR S22 OR S23 OR S24 | 1,049,588 |
| S26 | (MH "Quality of Life") | 153,190 |
| S27 | (MH "Personal Satisfaction") | 18,007 |
| S28 | TI ( ("quality of life" or wellbeing or "well being" or "life satis*") ) OR AB ( ("quality of life" or wellbeing or "well being" or "life satis*") ) | 244,527 |
| S29 | S26 OR S27 OR S28 | 306,352 |
| S30 | S25 AND S29 | 115,975 |
| S31 | ( MH "Ferrans and Powers Quality of Life Index" ) OR (MH "Family Member Well-Being Index") | 38,521 |
| S32 | TI ( (("Quality of Life" or QOL or HRQOL or EUROQOL or wellbeing or "well being" or (satisf* N1 life)) N3 (Index* or instrument* or scale* or assess* or questionnair* or measur* or survey* or test* or scheme* or tool* or scoring* or score*)) ) OR AB ( (("Quality of Life" or QOL or HRQOL or EUROQOL or wellbeing or "well being" or (satisf* N1 life)) N3 (Index* or instrument* or scale* or assess* or questionnair* or measur* or survey* or test* or scheme* or tool* or scoring* or score*)) ) | 58,593 |
| S33 | TI ( (WHOLQOL* or WHOQOL* or "WHO QOL*" or DEMQOL or EQ5D* or "EQ 5D*" or "European Quality of Life 5 Dimensions" or CarerQol* or "casp 19" or "scales measuring the impact of dementia on carers" or sidecar or "QOL-AD" or CQLI or "QOLLTI F" or ("dement* specific" N0 ("Quality of Life" or QOL or HRQOL))) ) OR AB ( (WHOLQOL* or WHOQOL* or "WHO QOL*" or DEMQOL or EQ5D* or "EQ 5D*" or "European Quality of Life 5 Dimensions" or CarerQol* or "casp 19" or "scales measuring the impact of dementia on carers" or sidecar or "QOL-AD" or CQLI or "QOLLTI F" or ("dement* specific" N0 ("Quality of Life" or QOL or HRQOL))) ) | 3,314 |
| S34 | S30 OR S31 OR S32 OR S33 | 168,573 |
| S35 | S3 AND S16 AND S34 | 1,770 |
| S36 | S3 AND S16 AND S34  Limiters - Publication Date: 20240101-20251231 | 87 |
| S37 | S3 AND S16 AND S34  Narrow by Language: - english (no publications in Scandinavian languages) | 86 |

**Database:** SocIndex (EbscoHost)

**Date:** 09.01.2025

**Results:** 10

| **#** | **Query** | **Results** |
| --- | --- | --- |
| S1 | DE "SENILE dementia" | 214 |
| S2 | DE "ALZHEIMER'S disease" | 2,581 |
| S3 | TI ( (dement* or senil* or presenil* or alzheimer*) ) OR AB ( (dement* or senil* or presenil* or alzheimer*) ) OR KW ( (dement* or senil* or presenil* or alzheimer*) ) OR SU ( (dement* or senil* or presenil* or alzheimer*) ) | 13,075 |
| S4 | S1 OR S2 OR S3 | 13,075 |
| S5 | DE "CAREGIVERS" | 8,735 |
| S6 | DE "MALE caregivers" | 61 |
| S7 | DE "CHILD caregivers" | 131 |
| S8 | DE "WOMEN caregivers" | 109 |
| S9 | DE "PSYCHOLOGY of caregivers" | 1,686 |
| S10 | DE "OLDER caregivers" | 23 |
| S11 | DE "HEALTH of caregivers" | 59 |
| S12 | DE "FAMILY relationships of caregivers" | 36 |
| S13 | DE "MEDICAL personnel-caregiver relationships" | 67 |
| S14 | DE "CAREGIVERS -- Social aspects" | 36 |
| S15 | DE "KINSHIP care" | 517 |
| S16 | TI ( (caregiver* or "care giver*" or carer* or caretaker* or "care taker*" ) ) OR AB ( (caregiver* or "care giver*" or carer* or caretaker* or "care taker*" ) ) OR KW ( (caregiver* or "care giver*" or carer* or caretaker* or "care taker*" ) ) OR SU ( (caregiver* or "care giver*" or carer* or caretaker* or "care taker*" ) ) | 25,833 |
| S17 | S5 OR S6 OR S7 OR S8 OR S9 OR S10 OR S11 OR S12 OR S13 OR S14 OR S15 OR S16 | 26,070 |
| S18 | DE "FAMILIES" OR DE "FAMILY relations" OR DE "FAMILY relationships of older people" OR DE "FAMILIES of people with disabilities" OR DE "ADULT children" OR DE "ADULT children of aging parents" OR DE "PARENT-adult child relationships" OR DE "ADULT children living with parents" OR DE "FAMILY relationships of adult children of aging parents" OR DE "ADULT children family relationships" | 50,582 |
| S19 | DE "DAUGHTERS" OR DE "NUCLEAR families" OR DE "PARENTS" OR DE "SIBLINGS" OR DE "SONS" OR DE "BROTHERS" OR DE "SISTERS" OR DE "HUSBANDS" OR DE "WIVES" OR DE "SIGNIFICANT others" OR DE "MARRIED people" OR DE "SPOUSES" OR DE "MARRIAGE" OR DE "MARITAL relations" OR DE "mothers" OR DE "fathers" | 46,786 |
| S20 | S18 OR S19 | 88,771 |
| S21 | TI ( (caring or caregiving or "care giving") ) OR AB ( (caring or caregiving or "care giving") ) OR KW ( (caring or caregiving or "care giving") ) OR SU ( (caring or caregiving or "care giving") ) | 20,616 |
| S22 | S20 AND S21 | 3,172 |
| S23 | TI ( ((child* or son or sons or daughter* or sibling* or brother* or sister* or wife* or wives or husband* or partner* or spous* or married* or famil* or parent* or father* or mother* or "next of kin*" or kinship* or "significant other*" or relative* or informal or unpaid) N2 (care or caring or caregiving or "care giving")) ) OR AB ( ((child* or son or sons or daughter* or sibling* or brother* or sister* or wife* or wives or husband* or partner* or spous* or married* or famil* or parent* or father* or mother* or "next of kin*" or kinship* or "significant other*" or relative* or informal or unpaid) N2 (care or caring or caregiving or "care giving")) ) OR KW ( ((child* or son or sons or daughter* or sibling* or brother* or sister* or wife* or wives or husband* or partner* or spous* or married* or famil* or parent* or father* or mother* or "next of kin*" or kinship* or "significant other*" or relative* or informal or unpaid) N2 (care or caring or caregiving or "care giving")) ) OR SU ( ((child* or son or sons or daughter* or sibling* or brother* or sister* or wife* or wives or husband* or partner* or spous* or married* or famil* or parent* or father* or mother* or "next of kin*" or kinship* or "significant other*" or relative* or informal or unpaid) N2 (care or caring or caregiving or "care giving")) ) | 36,847 |
| S24 | S17 OR S22 OR S23 | 56,410 |
| S25 | DE "SELF-evaluation" | 10,995 |
| S26 | TI ( ((caregiver* or "care giver*" or carer*) N3 (Index* or instrument* or scale* or assessment* or questionnair* or measur* or survey* or test or tests or scheme* or tool* or scoring* or score*)) ) OR AB ( ((caregiver* or "care giver*" or carer*) N0 (Index* or instrument* or scale* or assessment* or questionnair* or measur* or survey* or test or tests or scheme* or tool* or scoring* or score*)) ) OR KW ( ((caregiver* or "care giver*" or carer*) N3 (Index* or instrument* or scale* or assessment* or questionnair* or measur* or survey* or test or tests or scheme* or tool* or scoring* or score*)) ) OR SU ( ((caregiver* or "care giver*" or carer*) N3 (Index* or instrument* or scale* or assessment* or questionnair* or measur* or survey* or test or tests or scheme* or tool* or scoring* or score*)) ) | 486 |
| S27 | TI ( (dement* N1 (Index* or instrument* or scale* or assessment* or questionnair* or measur* or survey* or test or tests or scheme* or tool* or scoring* or score*)) ) OR AB ( (dement* N1 (Index* or instrument* or scale* or assessment* or questionnair* or measur* or survey* or test or tests or scheme* or tool* or scoring* or score*)) ) OR KW ( (dement* N1 (Index* or instrument* or scale* or assessment* or questionnair* or measur* or survey* or test or tests or scheme* or tool* or scoring* or score*)) ) OR SU ( (dement* N1 (Index* or instrument* or scale* or assessment* or questionnair* or measur* or survey* or test or tests or scheme* or tool* or scoring* or score*)) ) | 539 |
| S28 | TI ( ("sf 6" or "short form 6" or "sf 8"or "short form 8" or "sf 12" or "Short Form 12" or "sf 20" or "short form 20" or "sf 36" or "short form 36" or "Medical outcomes study short-form health survey") ) OR AB ( ("sf 6" or "short form 6" or "sf 8"or "short form 8" or "sf 12" or "Short Form 12" or "sf 20" or "short form 20" or "sf 36" or "short form 36" or "Medical outcomes study short-form health survey") ) OR KW ( ("sf 6" or "short form 6" or "sf 8"or "short form 8" or "sf 12" or "Short Form 12" or "sf 20" or "short form 20" or "sf 36" or "short form 36" or "Medical outcomes study short-form health survey") ) OR SU ( ("sf 6" or "short form 6" or "sf 8"or "short form 8" or "sf 12" or "Short Form 12" or "sf 20" or "short form 20" or "sf 36" or "short form 36" or "Medical outcomes study short-form health survey") ) | 166 |
| S29 | S25 OR S26 OR S27 OR S28 | 12,133 |
| S30 | DE "QUALITY of life" OR DE "WELL-being" | 30,352 |
| S31 | DE "SATISFACTION" | 7,385 |
| S32 | TI ( ("quality of life" or wellbeing or "well being" or "life satis*") ) OR AB ( ("quality of life" or wellbeing or "well being" or "life satis*") ) OR KW ( ("quality of life" or wellbeing or "well being" or "life satis*") ) OR SU ( ("quality of life" or wellbeing or "well being" or "life satis*") ) | 73,023 |
| S33 | S30 OR S31 OR S32 | 77,067 |
| S34 | S29 AND S33 | 1,605 |
| S35 | DE "QUALITY of life measurement" | 74 |
| S36 | TI ( (("Quality of Life" or QOL or HRQOL or EUROQOL or wellbeing or "well being" or (satisf* N1 life)) N3 (Index* or instrument* or scale* or assess* or questionnair* or measur* or survey* or test* or scheme* or tool* or scoring* or score*)) ) OR AB ( (("Quality of Life" or QOL or HRQOL or EUROQOL or wellbeing or "well being" or (satisf* N1 life)) N3 (Index* or instrument* or scale* or assess* or questionnair* or measur* or survey* or test* or scheme* or tool* or scoring* or score*)) ) OR KW ( (("Quality of Life" or QOL or HRQOL or EUROQOL or wellbeing or "well being" or (satisf* N1 life)) N3 (Index* or instrument* or scale* or assess* or questionnair* or measur* or survey* or test* or scheme* or tool* or scoring* or score*)) ) OR SU ( (("Quality of Life" or QOL or HRQOL or EUROQOL or wellbeing or "well being" or (satisf* N1 life)) N3 (Index* or instrument* or scale* or assess* or questionnair* or measur* or survey* or test* or scheme* or tool* or scoring* or score*)) ) | 9,107 |
| S37 | TI ( (WHOLQOL* or WHOQOL* or "WHO QOL*" or DEMQOL or EQ5D* or "EQ 5D*" or "European Quality of Life 5 Dimensions" or CarerQol* or "casp 19" or "scales measuring the impact of dementia on carers" or sidecar or "QOL-AD" or CQLI or "QOLLTI F" or ("dement* specific" N0 ("Quality of Life" or QOL or HRQOL))) ) OR AB ( (WHOLQOL* or WHOQOL* or "WHO QOL*" or DEMQOL or EQ5D* or "EQ 5D*" or "European Quality of Life 5 Dimensions" or CarerQol* or "casp 19" or "scales measuring the impact of dementia on carers" or sidecar or "QOL-AD" or CQLI or "QOLLTI F" or ("dement* specific" N0 ("Quality of Life" or QOL or HRQOL))) ) OR KW ( (WHOLQOL* or WHOQOL* or "WHO QOL*" or DEMQOL or EQ5D* or "EQ 5D*" or "European Quality of Life 5 Dimensions" or CarerQol* or "casp 19" or "scales measuring the impact of dementia on carers" or sidecar or "QOL-AD" or CQLI or "QOLLTI F" or ("dement* specific" N0 ("Quality of Life" or QOL or HRQOL))) ) OR SU ( (WHOLQOL* or WHOQOL* or "WHO QOL*" or DEMQOL or EQ5D* or "EQ 5D*" or "European Quality of Life 5 Dimensions" or CarerQol* or "casp 19" or "scales measuring the impact of dementia on carers" or sidecar or "QOL-AD" or CQLI or "QOLLTI F" or ("dement* specific" N0 ("Quality of Life" or QOL or HRQOL))) ) | 352 |
| S38 | S34 OR S35 OR S36 OR S37 | 10,438 |
| S39 | S4 AND S24 AND S38 | 163 |
| S40 | S4 AND S24 AND S38 | 163 |
| S41 | S4 AND S24 AND S38  Limiters - Peer Reviewed,  Date: 20240101-20251231 | 10 |

**Database**: Web of Science

**Date**: 09.01.2025

**Results**: 122

| # | Search Query | Results |
| --- | --- | --- |
| 1 | TS=(dement* or senil* or presenil* or alzheimer*) | 438219 |
| 2 | TS=((child* or son or sons or daughter* or sibling* or brother* or sister* or wife* or wives or husband* or partner* or spous* or married* or famil* or parent* or father* or mother* or "next of kin*" or kinship* or "significant other*" or relative* or informal or unpaid) NEAR/2 (care or caring or caregiving or "care giving") ) | 115337 |
| 3 | TS=(caregiver* or "care giver*" or caretaker* or "care taker*" or carer*) | 150998 |
| 4 | #2 OR #3 | 244885 |
| 5 | #4 AND #1 | 25444 |
| 6 | TS=((caregiver* or "care giver*" or carer*) NEAR/0 (Index* or instrument* or scale* or assessment* or questionnair* or measur* or survey* or test or tests or scheme* or tool* or scoring* or score*) ) | 2858 |
| 7 | TI=((caregiver* or "care giver*" or carer*) NEAR/3 (Index* or instrument* or scale* or assessment* or questionnair* or measur* or survey* or test or tests or scheme* or tool* or scoring* or score*) ) | 2186 |
| 8 | TI=(dement* NEAR/1 (Index* or instrument* or scale* or assessment* or questionnair* or measur* or survey* or test or tests or scheme* or tool* or scoring* or score*) ) | 1729 |
| 9 | TS=("sf 6" or "short form 6" or "sf 8" or "short form 8" or "sf 12" or "Short Form 12" or "sf 20" or "short form 20" or "sf 36" or "short form 36" or "Medical outcomes study short-form health survey") | 44450 |
| 10 | #6 OR #7 OR #8 OR #9 | 50553 |
| 11 | TS=("quality of life" or wellbeing or "well being" or "life satis*") | 804470 |
| 12 | #11 AND #10 | 33931 |
| 13 | TS=(("Quality of Life" or QOL or HRQOL or EUROQOL or wellbeing or "well being" or (satisf* NEAR/1 life) ) NEAR/3 (Index* or instrument* or scale* or assess* or questionnair* or measur* or survey* or test* or scheme* or tool* or scoring* or score*) ) | 151453 |
| 14 | TS=(WHOLQOL* or WHOQOL* or "WHO QOL*" or DEMQOL or EQ5D* or "EQ 5D*" or "European Quality of Life 5 Dimensions" or CarerQol* or "casp 19" or "scales measuring the impact of dementia on carers" or sidecar or "QOL-AD" or CQLI or "QOLLTI F" or ("dement* specific" NEAR/0 ("Quality of Life" or QOL or HRQOL) )) | 23437 |
| 15 | #12 OR #13 OR #14 | 175498 |
| 16 | #15 AND #5 | 1552 |
| 17 | #15 AND #5 Timespan: 2024-01-01 to 2025-12-31 | 123 |
| 18 | #15 AND #5 and English (Languages) Timespan: 2024-01-01 to 2025-12-31 | 122 |

**Database**: Epistemonikos

**Date**: 09.01.2025

**Results**: 10

**Limitation:** Publication type: Systematic review. Publication year: 2024-2025

(title:(dement* OR senil* OR presenil* OR alzheimer*) OR abstract:(dement* OR senile* OR presenile* OR alzheimer*)) AND (title:(caregiver* OR "care giver" OR "care givers" OR carer* OR caretaker* OR "care taker" OR "care takers") OR abstract:(caregiver* OR "care giver" OR "care givers" OR carer* OR caretaker* OR "care taker" OR "care takers")) AND (title:(Index* OR instrument* OR scale* OR assessment* OR questionnair* OR measur* OR survey* OR test OR tests OR scheme* OR tool* OR scoring* OR score*) OR abstract:(Index* OR instrument* OR scale* OR assessment* OR questionnair* OR measur* OR survey* OR test OR tests OR scheme* OR tool* OR scoring* OR score*)) AND (title:("quality of life" OR wellbeing OR "well being" OR "life satisfaction") OR abstract:("quality of life" OR wellbeing OR "well being" OR "life satisfaction"))
