# Supplementary material for: Assessing quality of life—a scoping review of studies presenting quality of life instruments for informal caregivers of persons with dementia
Source: BMC Geriatr. 2025 Nov 22;25:976. doi: 10.1186/s12877-025-06455-x (PMC12661884; doi:10.1186/s12877-025-06455-x)
Supplement: Supplementary file 2 — Supplementary Material 2. [file 12877_2025_6455_MOESM2_ESM.docx]

**Supplementary File 3.** An example of the critical appraisal checklist for analytical cross-sectional studies included in the scoping review

The JBI Critical Appraisal Checklist for Analytical Cross-Sectional Studies is a tool developed by the Joanna Briggs Institute (JBI) to help researchers, reviewers, and clinicians systematically evaluate the methodological quality and risk of bias in analytical cross-sectional studies.

The checklist includes eight key questions, each targeting specific aspects of the study design, conduct, and reporting:

1. **Were the criteria for inclusion in the sample clearly defined?**
2. **Were the study subjects and the setting described in detail?**
3. **Was the exposure measured in a valid and reliable way?**
4. **Were objective, standard criteria used for measurement of the condition?**
5. **Were confounding factors identified?**
6. **Were strategies to deal with confounding factors stated?**
7. **Were the outcomes measured in a valid and reliable way?**
8. **Was appropriate statistical analysis used?**

Each question is answered with “Yes”, “No”, “Unclear”, or “Not applicable”, and the checklist does not generate a score, but rather facilitates a structured judgment about the study’s trustworthiness and relevance.

54. Moola S, Munn Z, Tufanaru C, Aromataris E, Sears K, Sfetcu R, Currie M, Qureshi R, Mattis P, Lisy K, Mu P-F. Chapter 7: Systematic reviews of etiology and risk. In: Aromataris E, Munn Z (Eds.). JBI Manual for Evidence Synthesis. JBI, 2020. Available from <https://synthesismanual.jbi.global>.

| **Author(s), year, title, (country of study) [Ref.nr.]** | **Key quality criteria met** | **Summary/Comments** |
| --- | --- | --- |
| Alzaben et al., (2024). The Psychological Symptoms and Their Relationship to the Quality of Life Among Dementia Patients Caregivers. (Jordan) [50] | Inclusion criteria: Clearly defined.  Study subjects & setting: Adequately described.  Exposure measurement: Utilised validated tools for anxiety and depression.  Outcome measurement: Quality of life assessed using standard instruments.  Confounding factors: Not explicitly identified.  Strategies to deal with confounding factors: Not stated.  Outcome measurement validity: Yes  Statistical analysis: Appropriate. | The study used standardised tools to measure psychological symptoms and QoL. However, potential confounding factors (e.g., duration of caregiving, caregiver relationship) were not fully addressed in the analysis, limiting causal interpretation. |
| Anderson et al. (2022). A Comparative Analysis of Family Quality of Life Between Heterosexual and Sexual Minority Caregivers of People with Dementia (USA) [51] | Inclusion criteria: Clearly defined.  Study subjects & setting: Well-described.  Exposure measurement: Utilised psychosocial measures.  Outcome measurement: Family quality of life assessed with validated tools.  Confounding factors: Identified (e.g., demographic variables).  Strategies to deal with confounding factors: Statistical adjustments were made.  Outcome measurement validity: Yes.  Statistical analysis: Appropriate | The study effectively controlled for key demographic variables and used appropriate comparative methods. It provides high internal validity for a cross-sectional design, though generalizability may be limited due to sample characteristics. |
| Gumikiriza-Onoria et al. (2024). Psychological distress among family caregivers of persons with Alzheimer’s disease and related dementias in Uganda (Uganda) [52] | Inclusion criteria: Clearly defined.  Study subjects & setting: Adequately described.  Exposure measurement: Validated scales for psychological distress were used.  Outcome measurement: Quality of life assessed using standard instruments.  Confounding factors: Not explicitly identified.  Strategies to deal with confounding factors: Not stated.  Outcome measurement validity: Yes.  Statistical analysis: Appropriate | Cross-sectional design with validated measures used in a low-resource context. Valuable regional insight; however, limited adjustment for confounders and modest reporting of recruitment methods. |
| Hu et al. (2023). Associations between Affiliate Stigma and Quality of Life among Caregivers of Individuals with Dementia: Mediated Roles of Caregiving Burden and Psychological Distress (Taiwan) [53] | Inclusion criteria: Clearly defined.  Study subjects & setting: Adequately described.  Exposure measurement: Validated scales were used for affiliate stigma.  Outcome measurement: Quality of life assessed using standard instruments.  Confounding factors: Identified (e.g., caregiving burden, psychological distress).  Strategies to deal with confounding factors: Mediation analyses conducted.  Outcome measurement validity: Yes.  Statistical analysis: Appropriate | The mediation model was tested with validated instruments. The statistical approach is robust. The cross-sectional nature limits causal interpretation, though findings offer relevant psychosocial insight. |
| Ibrahim et al. (2024). Burden of care and quality of life among informal caregivers to Alzheimer patients in Egypt (Egypt) [78] | Inclusion criteria: Clearly defined.  Study subjects & setting: Well-described.  Exposure measurement: Utilised the Montgomery Borgatta Caregiver Burden Scale.  Outcome measurement: Health-Related Quality of Life Scale used.  Confounding factors: Identified (e.g., demographic variables).  Strategies to deal with confounding factors: Statistical adjustments were made.  Outcome measurement Validity: Yes.  Statistical analysis: Appropriate | A descriptive study using standard burden and QoL measures was conducted. This study is crucial for Egypt, but it lacks detailed control of confounders and comprehensive participant profiling. |
| Jeun et al. (2024). Impact of carer burden, social support, and gratitude on quality of life in families of older adults with dementia (South Korea) [54] | Inclusion criteria: Clearly defined.  Study subjects & setting: Adequately described.  Exposure measurement: Utilised structured self-report questionnaires.  Outcome measurement: Quality of life assessed using validated tools.  Confounding factors: Identified (e.g., caregiver burden, social support).  Strategies to deal with confounding factors: Hierarchical regression analysis conducted.  Outcome measurement Validity: Yes.  Statistical analysis: Appropriate. | Correlational design grounded in a strong theoretical framework. Use of validated tools and appropriate regression analysis. Limited by self-report data and cultural specificity. |
| Kalaitzaki et al. (2022). Dementia Family Carers’ Quality of Life and Their Perceptions About Care-receivers’ Dementia Symptoms: The Role of Resilience (Greece) [55] | Inclusion criteria: Clearly defined.  Study subjects & setting: Well-described.  Exposure measurement: Used validated scales for resilience and perceptions of dementia symptoms.  Outcome measurement: Quality of life assessed using standard instruments.  Confounding factors: Identified (e.g., caregiver demographics).  Strategies to deal with confounding factors: A mediation analysis was conducted.  Outcome measurement Validity: Yes.  Statistical analysis: Appropriate. | Insightful focus on resilience and symptom perception. Validated instruments and multivariate analysis were used. Cross-sectional constraints apply, but findings are thematically strong. |
| Kimura et al. (2021). Young- and Late-Onset Dementia: A Comparative Study of Quality of Life, Burden, and Depressive Symptoms in Caregivers (Brazil) [56] | Inclusion criteria: Clearly defined.  Study subjects & setting: Adequately described.  Exposure measurement: Utilised validated tools for assessing caregiver burden and depressive symptoms.  Outcome measurement: Quality of life assessed using standard instruments.  Confounding Factors: Identified (e.g., age of dementia onset).  Strategies to deal with confounding factors: Comparative analysis between groups conducted.  Outcome measurement validity: Yes.  Statistical analysis: Appropriate. | Comparative cross-sectional design with subgroup analysis. Validated instruments used to assess depression, burden, and QoL. Well-described methodology; findings limited by non-randomised sample and self-report bias. |
| Kim & Cha (2022). Effect of Perceived Stress on Health-Related Quality of Life among Primary Caregiving Spouses of Patients with Severe Dementia: The Mediating Role of Depression and Sleep Quality (South Korea) [66] | Inclusion criteria: Clearly defined.  Study subjects & setting: Well-described.  Exposure measurement: Validated scales were used for perceived stress.  Outcome measurement: Health-related quality of life assessed using standard instruments.  Confounding factors: Identified (e.g., depression, sleep quality).  Strategies to deal with confounding factors: Mediation analysis conducted.  Outcome measurement Validity: Yes.  Statistical analysis: Appropriate | The mediation model was well executed using validated psychological and QoL scales. The statistical approach was thorough. Limitations include self-report bias and lack of temporal causality due to the cross-sectional design. |
| Lucijanić et al. (2021). Predictors of Health-related Quality of Life in informal caregivers of dementia patients in Zagreb, Croatia, a cross-sectional study (Croatia) [57] | Inclusion criteria: Clearly defined.  Study subjects & setting: Adequately described. Exposure measurement: Utilised validated tools for assessing caregiver burden.  Outcome measurement: Health-related quality of life assessed using standard instruments.  Confounding factors: Identified (e.g., caregiver demographics).  Strategies to deal with confounding factors: Statistical adjustments were made.  Outcome measurement validity: Yes.  Statistical analysis: Appropriate | Cross-sectional study with regression analysis identifying key predictors of caregiver QoL. Use of validated scales. Useful insights, but the study lacks discussion of recruitment strategy and potential selection bias. |
| Monteiro et al. (2024).  Burden and quality of life of family caregivers of Alzheimer’s disease patients: the role of forgiveness as a coping strategy (Portugal) [58] | Inclusion criteria: Clearly defined.  Study subjects & setting: Well-described.  Exposure measurement: Validated scales were used for forgiveness and caregiver burden.  Outcome measurement: Quality of life assessed using standard instruments.  Confounding factors: Identified (e.g., caregiver age, distress levels).  Strategies to deal with confounding factors: Statistical adjustments were made.  Outcome measurement validity: Yes.  Statistical analysis: Appropriate. | Cross-sectional analysis of psychological factors affecting QoL. The use of validated instruments and mediation modelling strengthens the findings. Limited by a small sample and cultural context. |
| Morrison et al. (2020). The Impact of Risk and Resistance Factors on Quality of Life in Caregivers of Individuals with Dementia (USA) [59] | Inclusion criteria: Clearly defined.  Study subjects & setting: Adequately described.  Exposure measurement: Utilised validated tools for assessing risk and resistance factors.  Outcome measurement: Quality of life assessed using standard instruments.  Confounding factors: Identified (e.g., caregiver age, relationship to care recipient).  Strategies to deal with confounding factors: Statistical adjustments were made to account for these factors.  Outcome measurement validity: Yes.  Statistical analysis: Appropriate | Correlational design informed by a theoretical framework. The regression analysis was well executed. Confounding is handled appropriately, but the non-longitudinal nature of the study still limits the results. |
| Nasreen et al. (2024). Caregiver burden, mental health, quality of life and self-efficacy of family caregivers of persons with dementia in Malaysia: baseline results of a psychoeducational intervention study (Malaysia) [60] | Inclusion criteria: Clearly defined.  Study subjects & setting: Well-described.  Exposure measurement: Validated scales were used to assess caregiver burden and mental health.  Outcome measurement: Quality of life and self-efficacy assessed using standard instruments.  Confounding factors: Identified (e.g., caregiver demographics, caregiving context).  Strategies to deal with confounding factors: Statistical adjustments were made.  Outcome measurement validity: Yes.  Statistical analysis: Appropriate. | Quasi-experimental baseline findings using validated mental health and QoL tools. Clear sampling procedures and relevance to middle-income settings. Control procedures and longitudinal data pending. |
| Pereira et al. (2021). Contributors and Moderators of Quality of Life in Caregivers of Alzheimer´s Disease Patients (Portugal) [61] | Inclusion criteria: Clearly defined.  Study subjects & setting: Adequately described.  Exposure measurement: Utilised validated instruments. Outcome measurement: Quality of life assessed using the Quality of Life in Alzheimer’s Disease – Caregiver Version.  Confounding factors: Identified (e.g., employment status, caregiving hours, patient behaviour problems).  Strategies to deal with confounding factors: Statistical analyses were conducted to assess moderating effects.  Outcome measurement validity: Yes.  Statistical analysis: Appropriate | Cross-sectional study using moderation analysis and validated psychosocial measures. Methodologically sound with appropriate model testing; lacks longitudinal follow-up to assess causal relationships. |
| Pothiban et al. (2020). Quality of life and the associated factors among family caregivers of older people with dementia in Thailand (Thailand) [61] | Inclusion criteria: Clearly defined.  Study subjects & setting: Adequately described.  Exposure measurement: Utilised structured questionnaires assessing caregiving experiences and social support.  Outcome measurement: Quality of life assessed using the Quality of Life in Alzheimer’s Disease scale.  Confounding factors: Identified (e.g., caregiving duration, social support).  Strategies to deal with confounding factors: Statistical analyses were conducted to identify associated factors.  Outcome measurement validity: Yes.  Statistical analysis: Appropriate | Descriptive study with clearly defined variables and use of validated tools. Statistically appropriate; however, interpretation is constrained by the study’s observational, non-causal design. |
| Sittironnarit et al. (2020). Quality of life and subjective burden of primary dementia caregivers in Bangkok, Thailand (Thailand) [63] | Inclusion criteria: Clearly defined.  Study subjects & setting: Adequately described.  Exposure measurement: The Thai version of the Zarit Burden Interview was used to assess subjective burden.  Outcome measurement: The Pictorial Thai Quality of Life (PTQL) instrument assessed quality of life.  Confounding factors: Identified (e.g., education level, income, caregiving hours).  Strategies to deal with confounding factors: Multiple regression analyses were conducted to determine predictive factors.  Outcome measurement validity: Yes.  Statistical analysis: Appropriate | Cross-sectional survey using validated burden and QoL measures. Correlation-based findings are informative but cannot establish directionality. Cultural specificity is a strength and a limitation. |
| Silarova et al. (2023). Feasibility, validity and reliability of the ASCOT-Proxy and ASCOT-Carer among unpaid carers of people living with dementia in England (UK) [64] | Inclusion criteria: Clearly defined.  Study subjects & setting: Adequately described.  Exposure Measurement: Not applicable, as the study focused on validating measurement tools.  Outcome measurement: Assessed using the ASCOT-Proxy and ASCOT-Carer instruments.  Confounding factors: Not applicable.  Strategies to deal with confounding factors: Not applicable.  Outcome measurement validity: Yes; the study evaluated the psychometric properties of the instruments.  Statistical analysis: Appropriate; included Rasch analysis and construct validity assessments. | High-quality psychometric study with a focus on unpaid carers. Strong evidence for reliability and validity; proper in care evaluation frameworks. Broader use may require further contextual validation. |
| Wang et al. (2020). Job Demands and the Effects on Quality of Life of Employed Family Caregivers of Older Adults With Dementia: A Cross-Sectional Study (Taiwan) [65] | Inclusion criteria: Clearly defined.  Study subjects & setting: Adequately described.  Exposure measurement: Assessed job demands, including working hours, workplace inflexibility, and work inefficiency, using structured questionnaires.  Outcome measurement: Quality of life measured using appropriate instruments.  Confounding factors: Identified (e.g., demographic variables, caregiving resources).  Strategies to deal with confounding factors: Hierarchical multiple regression analyses were conducted to control for confounders.  Outcome measurement validity: Yes.  Statistical analysis: Appropriate | Cross-sectional survey grounded in job strain theory. Robust statistical controls were applied; self-report limitations were acknowledged. Substantial implications for work–caregiver role balance. |

**Summary table of JBI appraisals for cross-sectional studies**

| **Study** | **Country** | **Inclusion Criteria Clear** | **Subjects & Setting Described** | **Exposure Measured Validly** | **Outcome Measured Validly** | **Confounding Factors Identified** | **Strategies for Confounding Stated** | **Outcome Measurement Reliable** | **Statistical Analysis Appropriate** | **Overall Appraisal** |
| --- | --- | --- | --- | --- | --- | --- | --- | --- | --- | --- |
| Alzaben et al. (2024) | Jordan | Yes | Yes | Yes | Yes | No | No | Yes | Yes | Moderate |
| Anderson et al. (2022) | USA | Yes | Yes | Yes | Yes | Yes | Yes | Yes | Yes | High |
| Gumikiriza-Onoria et al. (2024) | Uganda | Yes | Yes | Yes | Yes | No | No | Yes | Yes | Moderate |
| Hu et al.  (2023) | Taiwan | Yes | Yes | Yes | Yes | Yes | Yes | Yes | Yes | High |
| Ibrahim et al. (2024) | Egypt | Yes | Yes | Yes | Yes | Yes | Yes | Yes | Yes | High |
| Jeun et al. (2024) | South Korea | Yes | Yes | Yes | Yes | Yes | Yes | Yes | Yes | High |
| Kalaitzaki et al. (2022) | Greece | Yes | Yes | Yes | Yes | Yes | Yes | Yes | Yes | High |
| Kimura et al. (2021) | Brazil | Yes | Yes | Yes | Yes | Yes | Yes | Yes | Yes | High |
| Kim & Cha (2022) | South Korea | Yes | Yes | Yes | Yes | Yes | Yes | Yes | Yes | High |
| Lucijanić et al. (2021) | Croatia | Yes | Yes | Yes | Yes | Yes | Yes | Yes | Yes | High |
| Monteiro et al. (2024) | Portugal | Yes | Yes | Yes | Yes | Yes | Yes | Yes | Yes | High |
| Morrison et al. (2020) | USA | Yes | Yes | Yes | Yes | Yes | Yes | Yes | Yes | High |
| Nasreen et al. (2024) | Malaysia | Yes | Yes | Yes | Yes | Yes | Yes | Yes | Yes | High |
| Pereira et al. (2021) | Portugal | Yes | Yes | Yes | Yes | Yes | Yes | Yes | Yes | High |
| Pothiban et al. (2020) | Thailand | Yes | Yes | Yes | Yes | Yes | Yes | Yes | Yes | Good |
| Sittironnarit et al. (2020) | Thailand | Yes | Yes | Yes | Yes | Yes | Yes | Yes | Yes | High |
| Silarova et al. (2023) | UK | Yes | Yes | N/A | Yes | N/A | N/A | Yes | Yes | High (Validation study) |
| Wang et al. (2020) | Taiwan | Yes | Yes | Yes | Yes | Yes | Yes | Yes | Yes | High |
